# Simultaneous Multiplex Genome Engineering *via* Accelerated Natural Transformation in *Bacillus subtilis*

**DOI:** 10.3389/fmicb.2021.714449

**Published:** 2021-08-17

**Authors:** Aihua Deng, Zhaopeng Sun, Tiantian Wang, Di Cui, Lai Li, Shuwen Liu, Fei Huang, Tingyi Wen

**Affiliations:** ^1^CAS Key Laboratory of Pathogenic Microbiology and Immunology, Institute of Microbiology, Chinese Academy of Sciences, Beijing, China; ^2^College of Life Sciences, University of Chinese Academy of Sciences, Beijing, China; ^3^China Innovation Academy for Green Manufacture, Chinese Academy of Sciences, Beijing, China; ^4^Zenbio Biotech Co., Ltd., Chengdu, China; ^5^Savaid Medical School, University of Chinese Academy of Sciences, Beijing, China

**Keywords:** multiplex genome engineering, natural genetic transformation, all-in-one vector, competency factor, recombinase, *Bacillus subtilis*

## Abstract

Multiplex engineering at the scale of whole genomes has become increasingly important for synthetic biology and biotechnology applications. Although several methods have been reported for engineering microbe genomes, their use is limited by their complex procedures using multi-cycle transformations. Natural transformation, involving in species evolution by horizontal gene transfer in many organisms, indicates its potential as a genetic tool. Here, we aimed to develop simultaneous multiplex genome engineering (SMGE) for the simple, rapid, and efficient design of bacterial genomes *via* one-step of natural transformation in *Bacillus subtilis*. The transformed DNA, competency factors, and recombinases were adapted to improved co-editing frequencies above 27-fold. Single to octuplet variants with genetic diversity were simultaneously generated using all-in-one vectors harboring multi-gene cassettes. To demonstrate its potential application, the tyrosine biosynthesis pathway was further optimized for producing commercially important resveratrol by high-throughput screening of variant pool in *B. subtilis*. SMGE represents an accelerated evolution platform that generates diverse multiplex mutations for large-scale genetic engineering and synthetic biology in *B. subtilis*.

## Introduction

Multiplex engineering at the scale of whole genomes has become increasingly important for biological research and biotechnological applications. Recent advancements in genetic manipulation have led to the development of several molecular biology techniques for complete multiplex genome editing, such as multiplexed automated genome engineering (MAGE; Wang et al., 2009), conjugative assembly genome engineering (CAGE; Isaacs et al., 2011), “coselection” MAGE (CoS-MAGE; Wang et al., 2012a), multiplex genome editing by natural transformation (MuGENT; Dalia et al., 2014), and RNA-guided nuclease technology based on the microbial clustered regularly interspaced short palindromic repeats adaptive immune system (CRISPR-Cas9/CRISPR-Cas12; Jiang et al., 2013; Mussolino and Cathomen, 2013; Wang et al., 2016; Westbrook et al., 2016; Qin et al., 2018; Liu et al., 2019). Although the CRISPR-Cas system has been used for multiplex gene editing, the selection of nucleases at the specific edited genomic loci limits its applications for producing complex mutant pools and accelerating cell evolution in microbial systems (Dalia et al., 2014; Nyerges et al., 2016). Utilizing highly efficient recombination with ssDNA oligonucleotides and multiple cycles of transformation, MAGE, CoS-MAGE, and CAGE have been used to introduce point mutations or small insertions/deletions into the genome of *Escherichia coli* for large-scale programming and evolution of cells (Wang et al., 2009; Isaacs et al., 2011; Wang et al., 2012a). However, these methods cannot be easily adapted for most microorganisms owing to low recombination efficiency and complex transformation procedure using successive multi-cycle transformations. Therefore, a simple, rapid, and high-efficiency transformation system has to be developed for use in a wide range of microorganisms.

As a well-characterized natural transformation system, *Bacillus subtilis* is well known for its ability to produce high levels of a wide range of biotechnology products, such as enzymes, antibiotics, vitamins, and nucleotides (Gu et al., 2018). Until now, site-specific recombination, counter-selectable markers, and the CRISPR-Cas systems have been used for gene modification in *B. subtilis* (Yan et al., 2008; Zhang et al., 2012; Bai et al., 2018; Wu et al., 2018; Liu et al., 2019). In previous studies, the use of site-specific recombination and counter-selectable methods could generate multiplex genetic variants only by multi-round transformation or multi-step modifications in a stepwise manner. Recently, CRISPR-based systems have achieved multiple point mutations (3–6) and in-frame knocking out of double genes in *B. subtilis* (Liu et al., 2019; Wu et al., 2020). However, the necessity of performing special selection for the protospacer adjacent motif sequences at the edited genomic loci limits the application of this method in large-scale genome engineering for accelerating evolution (Dalia et al., 2014; Nyerges et al., 2016). Therefore, an efficient multiplex genome engineering system is urgently required to implement large-scale genome editing in *B. subtilis* (Gu et al., 2018).

Natural genetic transformation (or natural transformation) occurs in a large number of organisms that can bind to and incorporate multiple double-strand DNA molecules from various environments (Lorenz and Wackernagel, 1994; Mell et al., 2011). This property has attracted considerable attention, as an extensive gene exchange network has been found to exist among microorganisms, plants, and animals, resulting in the evolution and adaptation of life in diverse environments (Lorenz and Wackernagel, 1994). Development of the competence state is triggered by the early, late, and delayed competence (*com*) factors, which might lead to the induction of more than 60 proteins involved in DNA uptake and processing of internalized DNA (Berge et al., 2017). Formation of natural competence varies in various microorganisms, and hence, two classic models, including the *Streptococcus*-*Bacillus* model (Gram-positive bacteria) and the *Haemophilus*-*Neisseria* model (Gram-negative bacteria), were established to understand the competent mechanisms in bacteria (Earl et al., 2008). The competent cells can integrate imported DNA into the genome *via* homologous recombination during transformation. This process contributes to a programmed mechanism of horizontal gene transfer in various organisms and plays an essential role in evolution and adaptation to various environments (Lorenz and Wackernagel, 1994; Earl et al., 2008; Serrano et al., 2018). Based on natural transformation, MuGENT was established to achieve multiplex genome editing in *Vibrio cholerae* and *Streptococcus pneumoniae* by multiple-cycles transformation of polymerase chain reaction (PCR) products harboring antibiotic markers or modified bases (Dalia et al., 2014). Therefore, natural transformation can be potentially applied as an efficient genetic tool, which could be exploited for multiplex genome engineering in *B. subtilis* with the superior ability of natural competence.

In this study, we aimed to develop a simultaneous multiplex genome engineering (SMGE) method to randomly modify multiple chromosomal loci in *B. subtilis via* one-step natural transformation. In contrast to previous methods, multi-round transformations or multi-step modifications are not needed. To demonstrate its potential application, the tyrosine biosynthesis pathway was firstly optimized in *B. subtilis* for producing commercially important chemicals. Therefore, SMGE might enable optimization of cellular metabolism and system-wide engineering for systems biology, synthetic biology, and evolutionary engineering in *B. subtilis*.

## Materials and Methods

### Bacterial Strains, Plasmids, Primers, and Culture Conditions

The bacterial strains and plasmids used in this study are listed in Supplementary Table S1. *Escherichia coli* EC135 lacking all known restriction-modification systems and orphan MTases was used to construct the recombinant plasmids (Zhang et al., 2012). Primer synthesis (Supplementary Table S2) and DNA sequencing were performed by Invitrogen (Shanghai, China), Tianyi Huiyuan (Beijing, China), and BGI (Beijing, China). The detailed information of all plasmids and strains constructed in this study was provided in Supplementary Materials. *E. coli* and *B. subtilis* were routinely cultured in the Luria-Bertani (LB) medium (10 g l^−1^ tryptone, 5 g l^−1^ yeast extract, and 5 g l^−1^ sodium chloride) at 37°C with appropriate antibiotics and chemicals. When appropriate, ampicillin (100 μg ml^−1^ for *E. coli*), kanamycin (10 μg ml^−1^ for *B. subtilis*), chloramphenicol (20 μg ml^−1^ for *E. coli* and 10 μg ml^−1^ for *B. subtilis*), tetracycline (20 μg ml^−1^ for *B. subtilis*), rifampicin (100 μg ml^−1^ for *B. subtilis*), spectinomycin (100 μg ml^−1^ for *B. subtilis*), and erythromycin (20 μg ml^−1^ for *B. subtilis*) were added to the medium.

### DNA Manipulation and Calculation of Transformation Efficiency

Naturally competent *B. subtilis* cells were prepared and transformed as follows using the conventional method. One fresh colony from the LB plate was inoculated in a 5-ml LB medium and cultured overnight. The culture (2%) was then transferred to a 15-ml freshly prepared nutritional deficiency (ND) medium containing 1 g l^−1^ casein hydrolysate. ND medium consists 9.29 g l^−1^ K_2_HPO_4_, 5.33 g l^−1^ KH_2_PO_4_, 0.78 g l^−1^ trisodium citrate, 20 g l^−1^ glucose, 0.05 g l^−1^ l-tryptophan, 0.01 g l^−1^ ferric ammonium citrate, 2 g l^−1^ potassium aspartate, and 0.36 g l^−1^ MgSO_4_. The culture was grown to OD_600_ = 1 and then continually shaken at 37°C for another 1 h. The culture was centrifuged at 8,000 × *g* for 10 min at 4°C and suspended in a 1.5-ml ND medium to obtain competent cells. For the transformation, 100 ng DNA and 400 μl ND medium were added to 100-μl competent cells. The mixture was incubated at 37°C for 20 min with agitation at 160 rpm. Then, 200 μl 2 × YT medium (16 g l^−1^ tryptone, 10 g l^−1^ yeast extract, and 5 g l^−1^ sodium chloride) was added into the mixture and incubated for 90 min.

High-recombinant *B. subtilis* cells were generated as follows. One fresh colony was inoculated in a 3-ml LB medium and cultured overnight. Then, 2-ml LB medium containing 2.5% (W/V) mannitol and xylose was supplemented to the overnight culture and continually incubated for another 1.5–2.0 h to induce competence. DNA was added into the competent cells and further incubated for 90 min.

The transformation mixtures were separately plated on LB medium with suitable selection to determine cellular viability, competence, and mutation efficiency. Considering that ∼10^8^ total viable cells were used per transformation, the transformation efficiency (CFU μg^−1^ DNA) was calculated as the number of colonies appearing on the antibiotic plates after the transformation of a microgram of plasmid DNA (pMK3 or pMK4; Zhang et al., 2012). Because random mutagenesis without coselection would be not feasible by the SMGE method, the method relied on selectable markers in multiplex genome editing design to screen multiple mutants with a simple and fast protocol. Thus, the co-editing frequency was calculated as the number of variants simultaneously harboring ≥2 mutations in all transformants grown on the antibiotic plate (= the number of multiple recombinants/total number of antibiotic-resistant recombinants; Dalia et al., 2014).

### Screening for Multiplex Mutants

To rapidly screen the recombinants, 15 endogenous genes (Supplementary Table S3) were selected as modified targets. In particular, *deoD* and *divIVA* were targeted for the knockout by introducing kanamycin and erythromycin resistance genes (*kan^R^* and *erm^R^*), respectively. Single base mutations were introduced into *tetL* and *ropB*, which conferred the mutants with tetracycline and rifampicin resistance, respectively. Eleven genes (*purA*, *thrC*, *ilvA*, *trpC*, *hisD*, *metA*, *lysA*, *recU*, *upp*, *amyE*, and *aprE*) were inactivated by introducing stop codon in the open reading frames. Among them, the inactivation of *purA*, *thrC*, *divIVA*, *trpC*, *hisD*, *metA*, and *lysA* rendered the mutants unable to synthesize adenosine, threonine, isoleucine, tryptophan, histidine, methionine, and lysine, respectively. In addition, inactivation of *recU*, *upp*, *amyE*, and *aprE* conferred loss of recombination repair activities, uracil phosphoribosyltransferase activity, starch hydrolysis activity, and protein hydrolysis activity, respectively.

To screen multiplex mutants, all recombinants from LB-Km plates were initially selected in the selective LB or minimal medium. LB media containing 10 μg ml^−1^ kanamycin (LB-Km), 50 μg ml^−1^ rifampicin (LB-Rif), 20 μg ml^−1^ erythromycin (LB-Erm), and 5 μg ml^−1^ tetracycline (LB-Tc) were used for screening *deoD*-inactive, *rpoB*-inactive, *divIVA*-inactive, and *tetL*-inactive mutants, respectively. LB media containing 100 μg ml^−1^ methyl methanesulfonate (LB-MMS), 10 mg ml^−1^ starch (LB-starch), and 10 mg ml^−1^ casein (LB-casein) were separately used for screening *recU*-inactive, *amyE*-inactive, and *aprE*-inactive mutants. MM media containing 10 μM 5-fluorouracil (MM-Fu), 10 mg ml^−1^ adenosine (MM-Ade), 10 mg ml^−1^ threonine (MM-Thr), 10 mg ml^−1^ tryptophan (MM-Trp), 10 mg ml^−1^ lysine (MM-Lys), 10 mg ml^−1^ histidine (MM-His), 10 mg ml^−1^ methionine (MM-Met), and 10 mg ml^−1^ isoleucine (MM-Ile) were used for screening *upp*-inactive, *purA*-inactive, *thrC*-inactive, *trpC*-inactive, *lysA*-inactive, *hisD*-inactive, *metA*-inactive, and *ilvA*-inactive mutants, respectively. After verification of the altered physiological characteristics using high-throughput 96-well or 48-well plates, the resulting multiplex variants were identified to determine the correct mutation sites by PCR and DNA sequencing using a set of primers binding upstream or downstream of the homologous sequences. Finally, the resistance genes in engineering strains were evicted to restore the markerless edited gene by transforming bacteria with the scarless genetic manipulation vectors (Supplementary Table S1).

### Fermentation in 48-Well Plates and Shake Flasks

*Bacillus subtilis* variants were cultured in seed medium for 12–16 h at 32°C and 200 rpm. The seed culture was then inoculated in 1-ml or 30-ml fermentation medium in 48-well plates or 500-ml shake flask at a ratio of 10% (v/v). Medium for fermentation was prepared as our previous study (Cui et al., 2019). Fermentation was performed at 36°C and 200 rpm for 85 h. The pH was controlled within the range of 6.4–7.0 by supplementing ammonia in the shake flask. Data show the averages of at least three independent experiments.

### Analysis of Product Concentration

Tyrosine concentration was quantified using High-Performance Liquid Chromatography (HPLC) (Agilent) with an Eclipse XDB-C18 column (4.6 × 150 mm; Agilent Technologies, United states). The eluent flow was at a constant rate of 1.0 ml min^−1^ with 80% reagent A (0.1% formic acid v/v) and 20% reagent B (methanol). The detection wavelength was set at 280 nm and the column was maintained at 30°C. The concentrations of tyrosine and resveratrol were calculated from the standard curves generated using the standards from Sigma, United States. The concentration of tyrosine and resveratrol was determined using HPLC according to our previous methods (Wang et al., 2018; Cui et al., 2019).

## Results

### High Co-Editing Frequency of All-in-One Plasmid DNA

**Figure 1:**
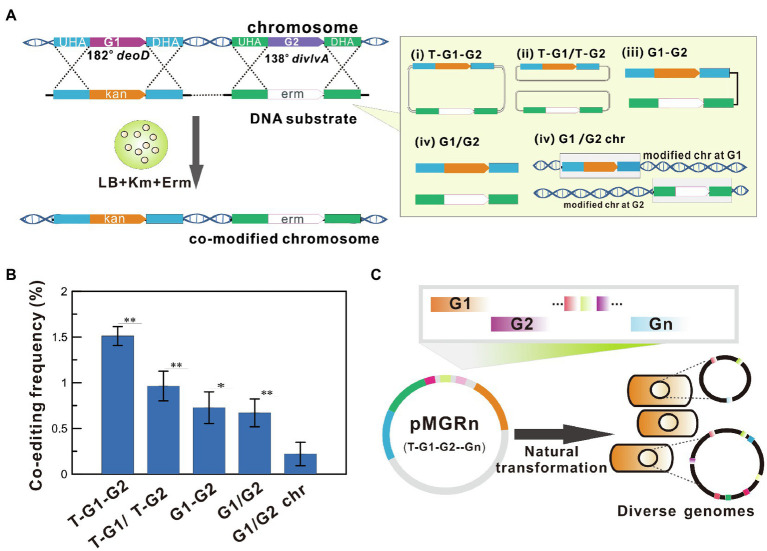
Simultaneously Multiplex Genome Engineering (SMGE) using an all-in-one recombination vector. **(A)** Diagram showing the approach used to determine co-editing frequency of allele replacement at two distant locations using different DNA forms. The all-in-one recombination vector harboring two target genes (T-G1-G2), a mixture of two different recombination vectors for each target gene (T-G1/T-G2), an all-in-one PCR product for two target genes (G1-G2), a mixture of two PCR products for each target gene (G1/G2), and a mixture of chromosomal DNA separately harboring integration of an antibiotic gene at the G1 or G2 site (G1/G2 chr) were used (see details in the Supplementary Materials). Luria-Bertani (LB) plates containing kanamycin or erythromycin were used to directly screen the single and double mutants. **(B)** The effects of different DNA construct on the co-editing frequency. All error bars indicate ± SD, *n* = 3. A value of *P* less than 0.05 was regarded to be a significant difference using the *T*-test (^*^p < 0.05; ^**^p < 0.01). **(C)** A schematic illustration of SMGE using an all-in-one vector harboring multi-gene recombination modules (pMGRn). G represents the targeted genetic locus.

To select the optimal DNA forms for multiplex genome engineering, the simultaneous editing frequency of co-modified mutants in all recombinants (called co-editing frequency) was investigated using natural transformation of different exogenous DNA forms into *B. subtilis*. As shown in Figure 1A, the recombinants that had simultaneously integrated *kan^R^* and *erm^R^* at two distant genomic loci (138^o^ and 182^o^) were rapidly screened on selective plates LB-Km and or/and LB-Erm. The results showed that the co-modified efficiency obtained using the plasmid as transformed DNA was higher than that of the PCR product (Figure 1B). The order of frequencies was T-G1-G2> T-G1/T-G2> G1-G2 > G1/G2> G1/G2 chr. Among these DNA constructs, the highest co-editing frequency of 1.51 ± 0.10% for simultaneous integration of two antibiotic resistance genes was obtained by transforming the all-in-one recombination vector pMGR2 (see the Supplementary Methods and Supplementary Table S1), which resulted in nearly a 0.5- to 5.8-fold increase over the frequencies obtained using other DNA forms (*p* < 0.01). In addition, chromosomal DNA displayed the lowest frequency owing to the difficulty of transforming genomic DNA molecules into cells. These results suggested that the all-in-one recombination vector exhibited the highest co-editing frequency in *B. subtilis* (Figure 1C). A mutant pool with diverse genomic mutations was generated by the transformation of a single plasmid harboring homologous arms of multiple target genes into the naturally competent *B. subtilis* cells.

**Figure 2:**
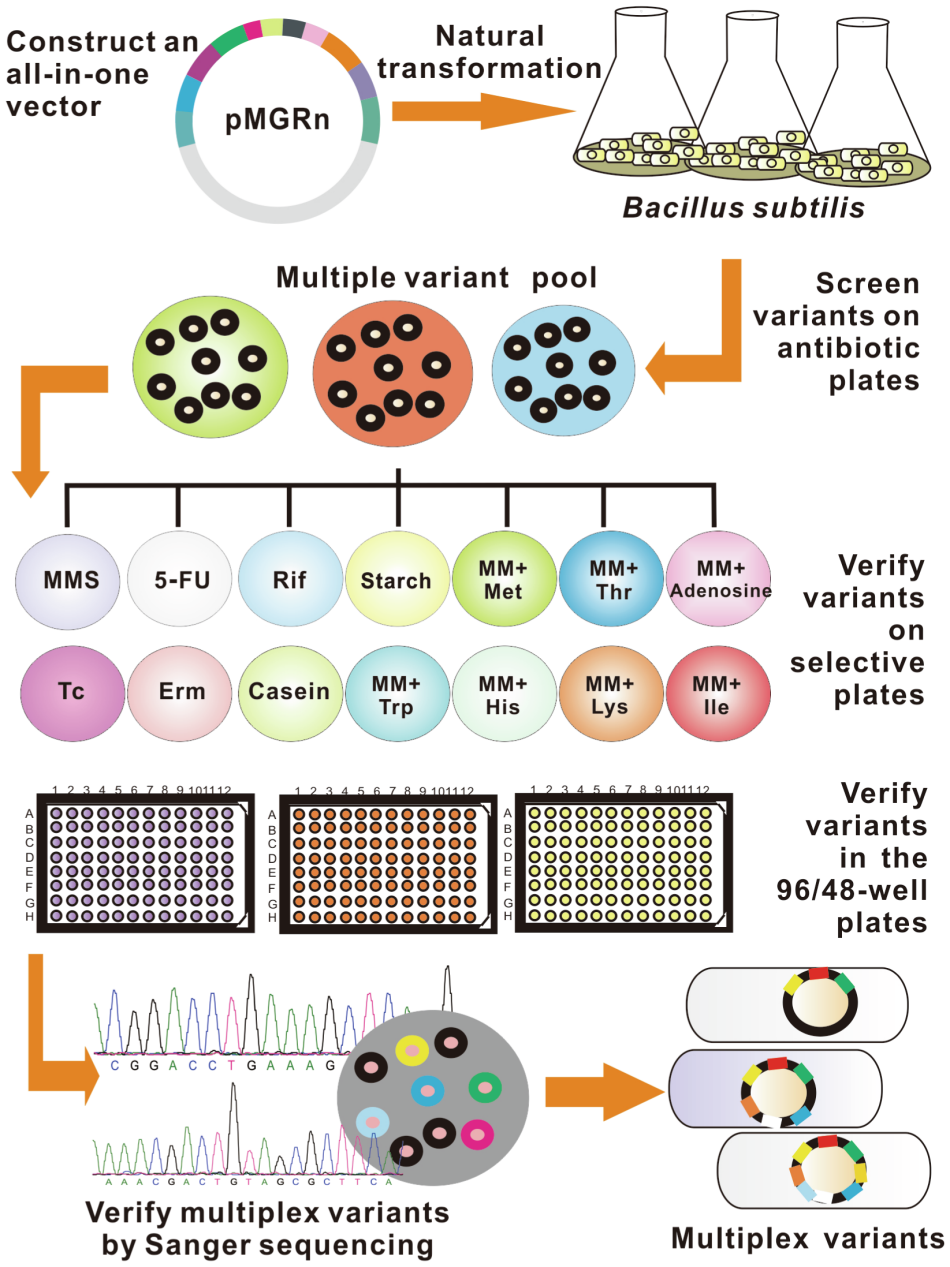
Detailed schematic showing SMGE. All variants were first screened in the Luria-Bertani (LB) plate containing antibiotics and then identified in the LB agar plates and 96-well/48-well plates containing selective media. Rif, Erm, Tc, and MMS represent LB plates containing rifampicin, erythromycin, tetracycline, and methyl methanesulfonate, respectively. 5-FU, starch, and casein represent LB media supplemented with 5-fluorouracil, starch, and casein, respectively. MM-Met, MM-Thr, MM-Ade, MM-Trp, MM-His, MM-Lys, and MM-Ile represent minimal media (MM) supplemented with methionine, threonine, adenosine, tryptophan, histidine, lysine, and isoleucine, respectively.

As a proof-of-concept of the study, the easily identified mutations were selected as the candidate targets for the preliminary experiment (see details in Materials and methods and Supplementary Materials). The detailed procedure of SMGE is as follows. First, an all-in-one plasmid (called pMGRn) was generated by assembling homologous arms of all target genes into the cloning T vector (TaKaRa, Dalian, China). Then, the mutant pool was generated after the transformation of pMGRn into the naturally competent cells. All variants were cultured on Luria-Bertani (LB) or minimal media (MM) plates containing various selective substrates to identify each mutant. Then, 96-well plates containing various selective media were used to confirm the positive mutants. Alternatively, 96-well (or 48-well) plates containing fermentation media can be used to verify the positive mutants designed for improving cellular performances *via* metabolic engineering. Finally, mutation sites were verified *via* PCR and DNA sequencing using a set of primers binding upstream or downstream of the homologous sequences to confirm the mutant pool with diverse genomes (Figure 2 and Supplementary Table S2).

### Simultaneous Modification of Three Genes Using pMGR6

**Figure 3:**
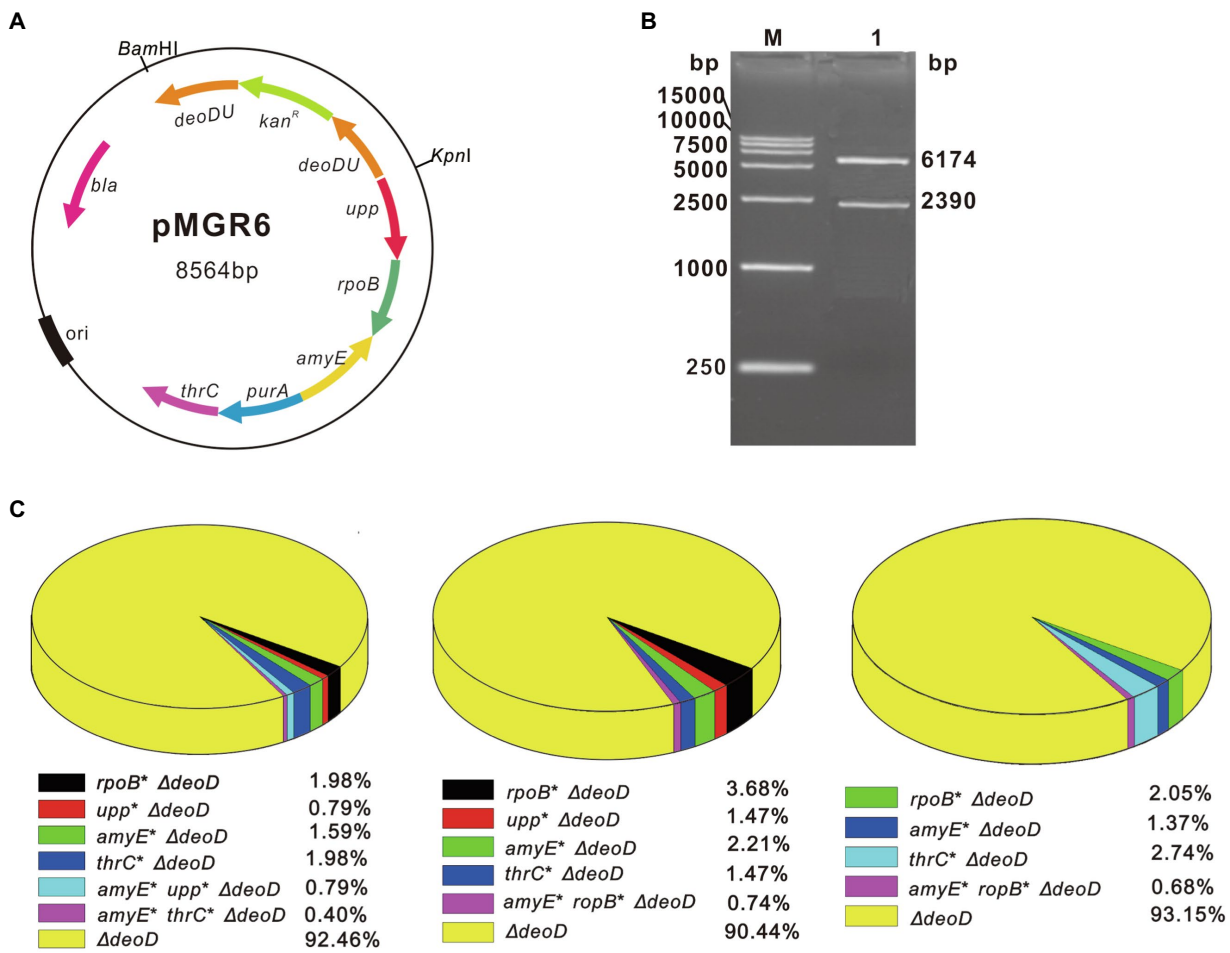
Co-editing frequencies of multiple variants using the SMGE method. **(A)** The map of the plasmid pMGR6. Homologous arms of six different target genes, *upp*, *ropB*, *amyE*, *deoD*, *purA*, and *thrC*, were inserted into the cloning T vector. (B) The verification of plasmid pMGR6 by digestion with restriction enzymes. The plasmid pMGR6 was verified by digestion with *Bam*HI and *Kpn*I. **(C)** The editing frequencies of three independent transformations of pMGR6. LB plates containing kanamycin (LB-Km) were used to rapidly select various recombinants when *deoD* was replaced by *kan^R^* in the chromosomal DNA.

To simultaneously modify multiple genetic loci using SMGE, the vector pMGR6 was constructed by ligating the homologous sequences of six different targets (*upp*, *ropB*, *amyE*, *deoD*, *purA*, and *thrC*) in the pMD19-T vector. After verification using restriction enzyme digestion and sequencing, pMGR6 was used as the recombination vector targeting six genomic loci (Figures 3A,B). *kan^R^* was integrated into the chromosomal DNA when *deoD* was knocked out by a double crossover event. Thus, LB-Km plates were used to rapidly select various recombinants after the natural transformation of pMGR6 into *B. subtilis*. Following the procedure shown in Figure 2, the mutant pool with ∼10^3^ colonies was screened in selective media. In three independent transforming experiments, single, double, and triple mutants with combinatorial genome modifications were generated (Figure 3C). Furthermore, the variants’ genotypes differed in these three experiments, demonstrating the randomness of the SMGE method. The co-editing frequencies of double and triple mutants in all kanamycin-resistant colonies (*∆deoD*:: *kan^R^*) were determined to be 0.79–3.68% and 0.40–0.79%, respectively. Therefore, up to three genes were successfully edited simultaneously by pMGR6 using the SMGE method.

### Rapid, Simple, and Efficient Simultaneous Modification of Four Genes in the Accelerated Natural Transformation System

**Figure 4:**
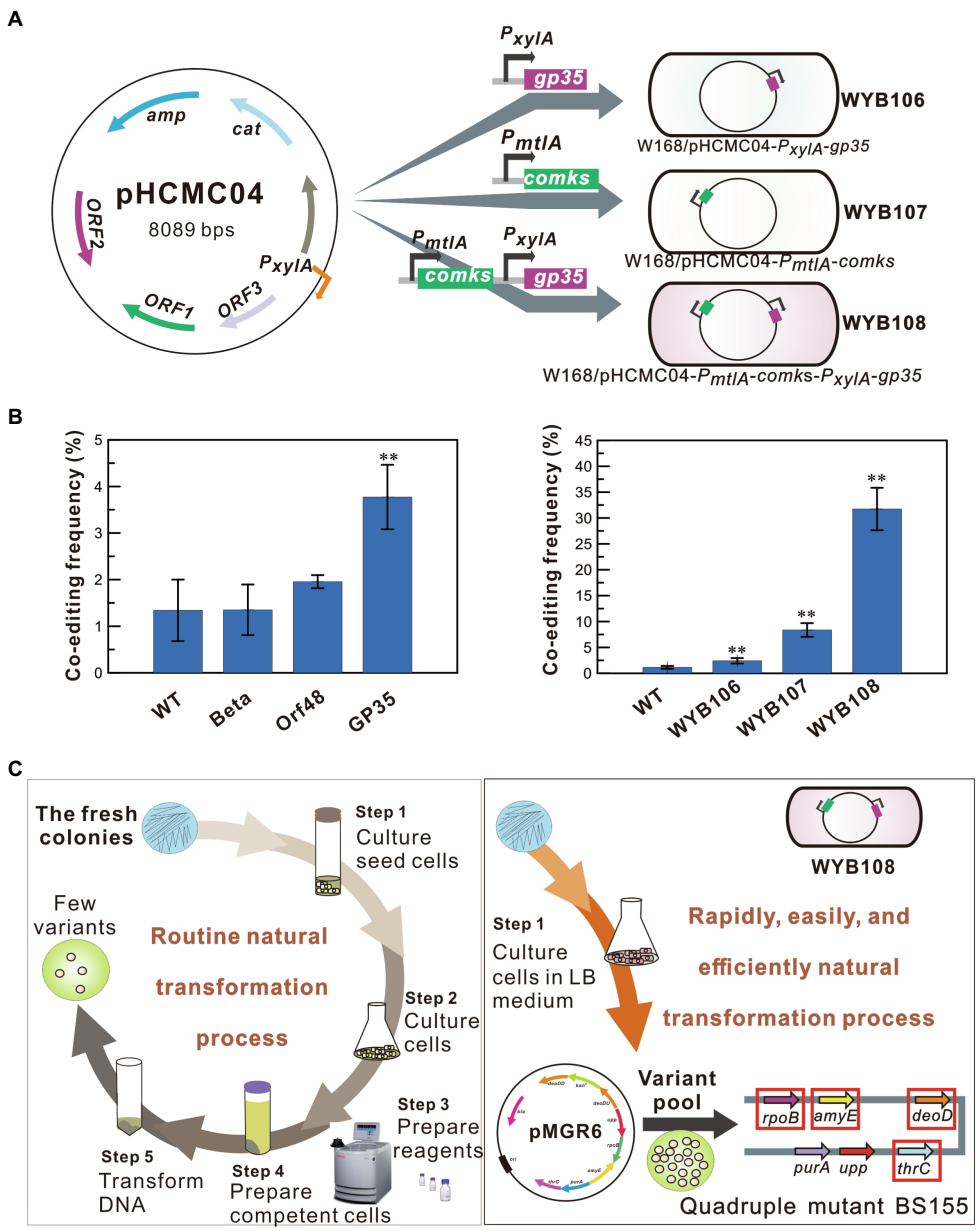
Enhancement of the co-editing frequency by recombinase and competency factor. **(A)** A schematic illustration of the high-recombinant strains generated by expressing the recombinase or/and the competency factor. **(B)** The co-editing frequencies of the high-recombinant strains expressing the recombinase or/and competency factor. All error bars indicate ± SD, *n* = 3. A value of *P* less than 0.05 was regarded to be a significant difference using the *T*-test (^**^p < 0.01). **(C)** Rapid and efficient simultaneous modification of four genetic loci in the high-recombinant strain WYB108 *via* simple natural transformation.

To further increase the multiplex editing efficiency of the SMGE method, the natural transformation system was separately optimized by introducing a recombinase and competency factor in *B. subtilis* (Figure 4A). Based on our previous recombination systems developed in *B. subtilis* (Sun et al., 2015), strains BS056, BS060, and BS061, separately expressing the exogenous recombinase genes *orf48*, *beta*, and *gp35* under the control of a xylose-inducible promoter, were selected to assay their effects on the co-editing frequencies (Figure 1A and Supplementary Table S2). The Orf48- and beta-expressing strains exhibited frequencies similar to that of the wild-type strain (WT). Notably, the recombinase GP35 from the native phage SPP1 showed the highest co-editing frequency in *B. subtilis*, exhibiting a 2.1-fold increase (*p* < 0.05) in efficiency compared to that of the WT strain (Figure 4B). Furthermore, the competency factors ComK and ComS also increased the co-editing frequency of *B. subtilis*, with a 7.3-fold increase (*p* < 0.001) over the WT strain (Figure 4B). Therefore, the genes encoding the recombinase GP35 and competency factors ComKS were all expressed in *B. subtilis* to construct the high-recombinant strain WYB108 (W168/pHCMC04-*comKS*-*gp35*). The editing frequencies of WYB108 increased significantly, which was 27.6-fold (*p* < 0.001) of that of the WT strain (Figure 4B). After the transformation of pMGR6 into the high-recombinant strain, mutant pools with ∼10^3^ colonies were screened following the procedure shown in Figure 2. As a result, a quadruple mutant, BS155 (WYB108∆deoD::kan^R^ ropB^*^ amyE^*^ thrC^*^), was generated (Figure 4C and Supplementary Figure S1). Therefore, SMGE significantly improved the co-editing frequency of natural transformation by more than 27-fold when suitable competency factors and recombinases were used, providing the possibility for the generation of a large number of genetically diverse mutant pools. More importantly, the preparatory steps for the transformation were further shortened to a rapid and easy culturing step in LB medium (Figure 4C and Supplementary Figure S2).

### Diverse Co-Modified Variants With Genetic Diversity Generated Using the SMGE Method

**Figure 5:**
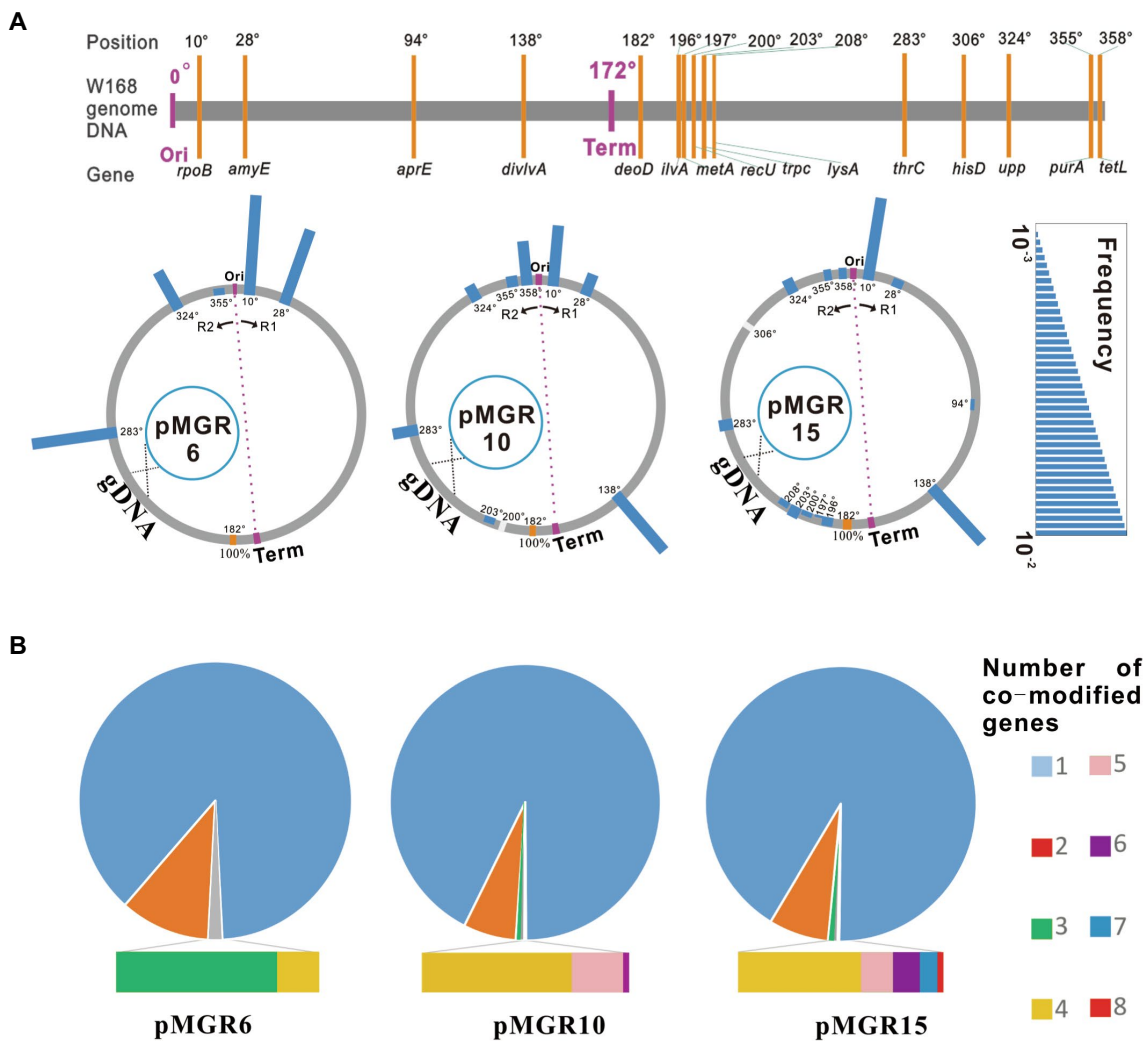
The co-editing frequency map and the number of co-modified genetic loci using the SMGE method. **(A)** Co-editing frequency maps of each targeted genetic locus across the *Bacillus subtilis* genome using different pMGR vectors. The position of each targeted genetic locus is marked in the *B. subtilis* genome. Positions of origin and terminus are shown as Ori and Term, respectively. R1 and R2 represent the two replication forks. gDNA represents the genome DNA. The scar bar represents the co-editing frequency. **(B)** Co-editing frequencies of multiplex variants using different pMGR vectors. The co-editing frequency was calculated as the number of desired variants in all colonies of the mutant pool. All measurements were performed in five independent experiments and average data were calculated. The original data have been supplemented in Supplementary Figure S4.

To determine the mutation frequency map and co-modified gene number of the mutant pool generated using the SMGE method, pMGR10 and pMGR15 harboring 10 and 15 targeted genes, respectively, were further constructed (see Supplementary Methods for details). Fifteen genes (*rpoB*, *amyE*, *aprE*, *divIVA*, *deoD*, *divIVA*, *metA*, *recU*, *trpC*, *lysA*, *thrC*, *hisD*, *upp*, *purA*, and *tetL*) distributed at various positions throughout the chromosomal DNA were selected as targets (Figure 5A). *deoD*, which was closest to the terminus, was used as the selective site by introducing *kan^R^*. The vectors pMGR6, pMGR10, and pMGR15 were separately transformed into the naturally competent cells. Mutant pools with ∼10^3^–10^5^ colonies were initially screened in various selective media (Figure 2). All variants with double and triple mutation sites were verified using PCR and DNA sequencing. Most targeted loci were modified with editing frequencies of 0.12–4.67%. However, the frequencies did not represent an obvious correlation with the chromosome position and with distance from the origin of replication (Ori) or the terminus (Term; Supplementary Figure S4). Notably, a high degree of variability, including single, double, triple, quadruple, quintuple, sextuple, septuple, and octuple mutants, was generated using SMGE (Figure 5B; Supplementary Figure S4). Quadruple, sextuple, and octuple mutations were separately generated using recombination vectors pMGR6, pMGR10, and pMGR15, with frequencies of 0.33, 0.04, and 0.02%, respectively. The diversity of the variants suggested that SMGE can be used as a multiplex engineering platform for introducing various mutations in the cell population.

### Optimization of the Tyrosine Biosynthesis Pathway for Resveratrol Production

**Figure 6:**
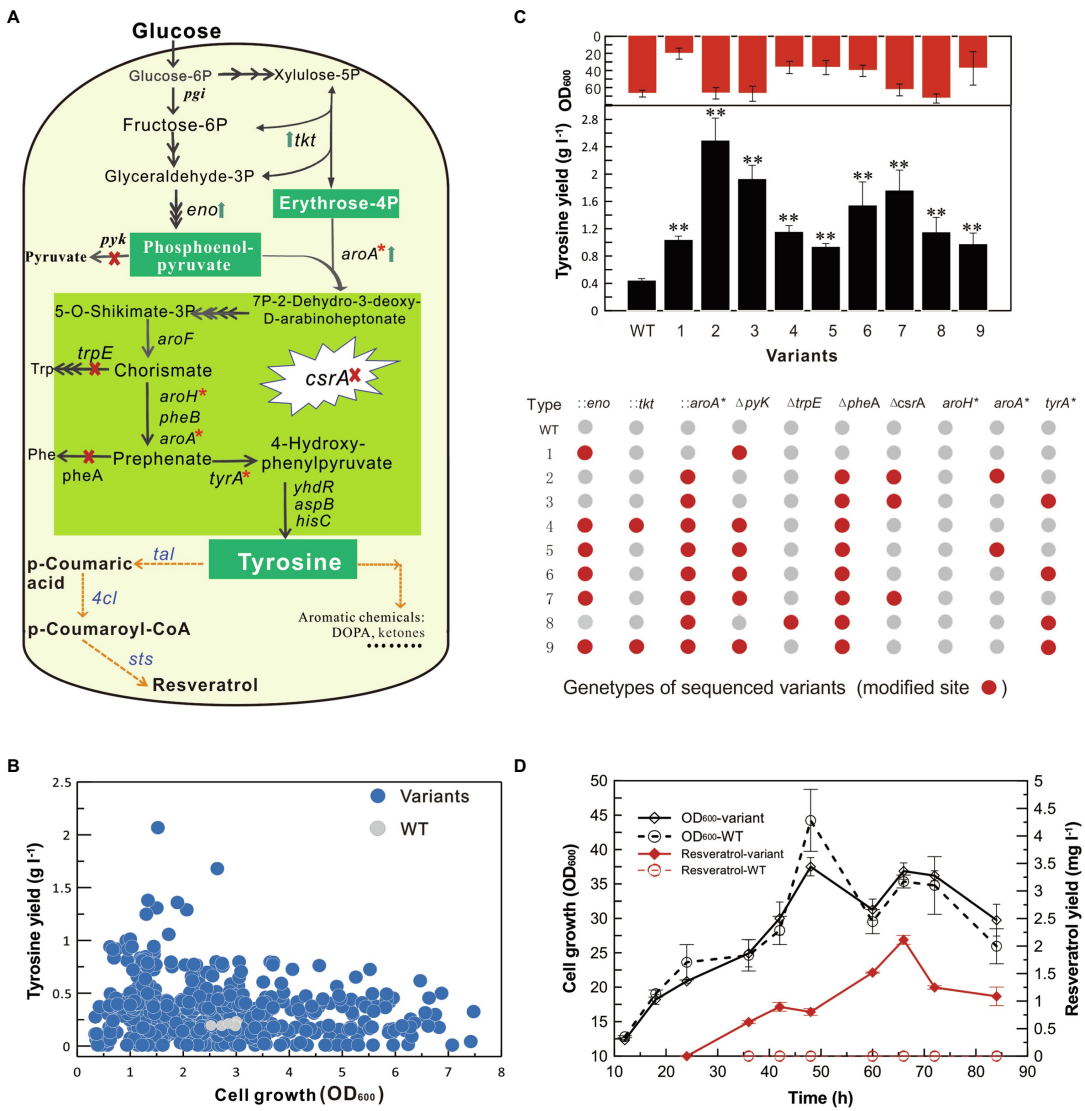
Optimization of the tyrosine biosynthesis pathway for resveratrol production. **(A)** Optimization of the tyrosine biosynthesis pathway in *B. subtilis*. Upward arrows represent the overexpression of targeted genes. Cross marks represent the knockout of target genes. Stars represent point mutations for eliminating feedback inhibition. **(B)** Production of tyrosine and cell growth of variants from the mutant pool generated using the SMGE method. Fermentation was performed in the high-throughput 48-well plates. **(C)** Modification of the tyrosine biosynthesis pathway of the optimized variants. Red points represent the modified genes, while the gray points represent the unmodified genes. WT represents the original *B. subtilis* W168 strain. **(D)** Cell growth and production of resveratrol. All error bars indicate ± SD, *n* = 3. The difference between each variant and WT was examined by the *T*-test and a value of P less than 0.05 was regarded to be a significant difference using (^**^p < 0.01).

To demonstrate the application of the SMGE method, we optimized the biosynthesis pathway of tyrosine, which is the precursor of different biochemical products, such as resveratrol, levodopa, salvianic acid A, and hydroxytyrosol. According to the tyrosine synthesis pathway in *B. subtilis*, the engineering strategy was designed as shown in Figure 6A. To enhance the precursors of phosphoenolpyruvate (PEP) and erythrose-4 phosphate (E4P), *tkt* and *eno* were overexpressed by integrating the expression cassettes into the chromosomal DNA. To further increase the condensation of E4P and PEP to form 3-deoxy-D-arabino-heptulosonate-7-phosphate (DAHP), the 5-enolpyruvylshikimate-3-phosphate synthase AroA in the first step of tyrosine synthesis pathway was overexpressed by integrating the feedback-resistant variant. Meanwhile, *pyk*, *trpE*, and *pheA* were knocked out to reduce the branch flux of the tyrosine synthesis. Additionally, *aroA*, *tyrA*, and *aroH* were targeted for point mutations to release feedback suppression. Finally, the transcriptional regulator CsrA was inactivated by disrupting the open reading frame to relieve the complex regulation of the PEP and tyrosine synthesis. The homologous arms of 10 targets (*tkt*, *eno*, *pyk*, *aroA*, *trpE*, *pheA*, *csrA*, tyrA^*^, aroH^*^, and aroA^*^) were assembled into the cloning T vector to construct the recombination vector pMGR-tyr10 (Figure 6A and Supplementary Figure S2). The detailed plasmid construction and modification sites were shown in the Supplementary Methods and Supplementary Table S2.

A mutant pool with diverse genomic mutations was generated on LB plates containing kanamycin, erythromycin, or spectinomycin after natural transformation of pMGR-tyr10 into the high-recombination strain. Up to 450 variants were randomly selected to be sub-cultured for 3 days (nine generations) in 96-well plates containing LB media to eliminate the GP35 and ComKS expression vector (pHCMC04-*comKS*-*gp35*; Supplementary Figure S7A), which were further used for detecting tyrosine production in 48-well plate fermentation. As shown in Figure 6B, several variants exhibited higher tyrosine production than the original WT strain. Among them, variants exhibiting more than 4-fold increase in tyrosine production were sequenced to determine the mutation sites (Supplementary Figure S7B). The sequence analysis classified variants into nine different genotypes that were further used for assessing tyrosine production in shake-flask fermentation (Figure 6C). The optimized variants exhibited as much as 0.5- to 4.0-fold increase in tyrosine production compared to that of the original WT strain. The tyrosine production has been significantly increased by overexpressing the key enzyme and eliminating the feedback inhibition of AroA^P192L^ in the first step of tyrosine biosynthesis, blocking phenylalanine synthesis pathway by deleting *pheA* gene, and relieving transcriptional regulation by inactivating carbon storage regulator (CsrA). Strain Type 2 (W168∆csrA ∆pheA:: aroA^*^), which showed the highest yield, was used as the chassis bacteria for the synthesis of resveratrol.

Because the native biosynthesis of resveratrol is not present in microbes, exogenous genes have to be selected from plants that are rich sources of resveratrol (Thapa et al., 2019). Based on the analysis of the metabolic pathway, *p*-scoumaric acid is first formed from either Phe or Tyr catalyzed by phenylalanine/tyrosine ammonia lyase (PAL/TAL). Then, the *p*-coumaric acid is esterified with coenzyme A (CoA) to form *p*-coumaroyl-CoA by the catalysis of 4-coumaroyl-CoA ligase (4CL). Finally, with the help of stilbene synthase (STS), *p*-coumaroyl-CoA is condensed with malonyl-CoA to produce resveratrol (Figure 6A). According to the alternative sources of resveratrol biosynthesis gene, *tal*, *4 cl*, and *sts* separately from *Herpetosiphon aurantiacus*, *Arabidopsis thaliana*, and *Vitis vinifera* have been produced a relatively high amount of resveratrol in yeast in our previous study (Wang et al., 2018). Thus, *tal*, *4 cl*, and *sts* were selected for resveratrol synthesis using tyrosine as the precursor, and the plasmid pWYE898 (pHCMC04^*^-*tal^BS^-P_veg_-4cl ^BS^- P_43_-sts^BS^*) was further transformed in the variant Type 2 and original W168 (WT) to obtain strains BS191 and BS192 (Supplementary Tables S1 and S2). The detailed processes of plasmid/strain construction and codon optimization were described in Supplementary Methods. Through flask cultivation of strains in 500-ml shake flask for 68 h, maximum 2.11 ± 0.08 mg l^−1^ resveratrol was detected in the fermentation products of BS192, which is the first report of resveratrol biosynthesis in *B. subtilis* (Figure 6D). However, no resveratrol was detected in the control strain BS191. After completion of genome engineering, the resistance genes were evicted to restore the markerless genome after transformation with the genetic manipulation vectors (Supplementary Table S1; Wu et al., 2018). Thus, SMGE was applied to optimize the tyrosine biosynthesis pathway for producing commercially important resveratrol from a genetically diverse mutant pool in a drastically shortened timescale, thereby providing a simple, easy, and effective approach for metabolic engineering and synthetic biology purposes.

## Discussion

In this study, the SMGE method was developed to generate multiple genetically diverse mutant pools using one-step natural transformation of an all-in-one vector. Until now, multiplex genome editing techniques have been used only in certain bacteria using multiple rounds of transformation. For example, MAGE has been used to manipulate the *E. coli* genome (Wang et al., 2009; Sandoval et al., 2012; Wang et al., 2012a). The technology was used to construct a large number of variants *via* successive 15-cycle transformations of 90-nucleotide oligos using an automated device, which realized the simultaneous modification of multiple targets in the chromosome (Isaacs et al., 2011; Lajoie et al., 2013). Owing to the short length of the homologous arm, the editing efficiency was extremely low in most microorganisms, and designs of gene deletions, insertions, and replacements were challenging (Dalia et al., 2014). In previous studies, the use of different lengths of oligonucleotides and recombinases was optimized to maximize the efficiency of gene editing in *B. subtilis*. Wang et al. reported that ssDNA recombination in *B. subtilis* requires a minimum of 70 nucleotide-long homology arms for DNA recombination (Wang et al., 2012b). Van and Hatfull reported low mutation efficiencies using short homologous arms in *Mycobacterium tuberculosis* (Van Kessel and Hatfull, 2007). Using PCR amplification of ssDNA with 500-bp homologous regions, we have developed a GP35-meditated recombination system with a high recombineering frequency in *B. subtilis* (Sun et al., 2015). However, we did not obtain multiplex genetic variants in the GP35-meditated recombination system using ssDNA as the transformed DNA by multiple rounds of transformation. In this case, an easy-to-operate natural transformation system was utilized to randomly modify multiple chromosomal loci in *B. subtilis* using one-step transformation of an all-in-one vector, including deletions, replacements, insertions, and point mutations.

Using PCR products harboring antibiotic markers or modified 6–30 bases, the MuGENT method shows that multiple rounds of natural transformation can produce multiple mutants in *V. cholerae* and *S. pneumoniae* (Dalia et al., 2014). In this study, an all-in-one vector was first adapted to generate multiplex mutant pools and simultaneously edit up to three genes *via* natural transformation (Figure 3). Furthermore, the recombinase GP35 and competency factors ComKS were introduced to simplify the manipulation procedure and increase the multiplex editing efficiency of the SMGE method (Figure 4). ComK is a global regulatory factor for natural transformation, which can initiate an auto-stimulatory response for its transcription and translation and start the transcription of late competent genes (Smits et al., 2005; Gamba et al., 2015). Alternatively, inactivation of the ComK protein will lead to the disappearance of the competent state (Vansinderen et al., 1995). During the development of competence, ComK is also targeted for degradation by ClpCP. A small protein, ComS, protects ComK from proteolysis during the stationary phase (Turgay et al., 1998). Therefore, ComK and ComS play a vital role in bacterial natural transformation, which can significantly increase the transformation efficiency and co-editing frequency due to overexpression of the two proteins that promote natural competence in *B. subtilis* (Figure 4B and Supplementary Figure S2A). In addition, GP35 effectively interacts with the DNA helicase (DnaC) and single-strand-binding protein (SsbA), promoting/catalyzing DNA strand invasion and exchange and eventually resulting in the activation of recombination in *B. subtilis* (Ayora et al., 2002). In a previous report, *comk* was overexpressed to simplify the manipulation procedure and improve the transformation efficiency, which was used to construct a mutant pool expressing cellulose from a plasmid in *B. subtilis* SCK6 (Zhang and Zhang, 2011). However, the competent factors were not utilized in that study to enhance editing efficiency and produce mutant pools for multiple genome editing. In this study, the co-editing frequency in the preliminary experiment was 1.51 ± 0.10% (Figure 1B), which is consistent with those of the double mutants (0.79–3.68%) and triple mutants (0.40–0.79%) using pMGR6 in the WT strain (Figure 3). After the introduction of the recombinase and competent factors, the frequencies increased to 31.74 ± 4.11%, which was significantly higher than that of the WT strain. The co-editing frequencies were 10.67, 1.33, and 0.33% for the double, triple, and quadruple mutants, respectively, using pMGR6 in the optimized strain (Figure 5), which showed improvement and advantage over the WT strain. Upon using the competency factors ComKS and recombinase GP35, the co-editing frequency increased by 27.6-fold in optimized natural transformation using the SMGE method, indicating the possibility of generating a large number of genetically diverse mutant pools (Figure 4). As a result, a high degree of variability (double, triple, quadruple, quintuple, sextuple, septuple, and octuple mutants) was generated *via* one-step natural transformation of vector (Figure 5). Compared to conventional natural transformation, which is time-consuming and inefficient, the optimized natural transformation system is simple, rapid, and efficient and can be performed by cultivating bacteria for 1.5–2.0 h in LB media after induction with competent factors (Figure 4C and Supplementary Figure S2; Bott and Wilson, 1967; Rahmer et al., 2015). Therefore, improved efficiency of natural transformation using SMGE indicated the superiority of this method as a multiplex genome engineering tool for large-scale genome engineering or directed evolution in *B. subtilis* (Dalia et al., 2014). In addition, the approach developed here could be applied in other bacterial species with similar capability of natural competence as *B. subtilis* (approximately 10^−2^ transformation frequency), such as *Thiobacillus*, *Azotobacter vinelandii*, *Thermus thermophiles*, *Neisseria meningitidis*, *Streptococcus*, and so on (Lorenz and Wackernagel, 1994).

To further investigate the potential relationship of the co-editing frequencies and the number of co-modified targets using the SMGE method, the plasmids pMGR6, pMGR10, and pMGR15 were separately used to generate diverse genetic variants. Almost all the target loci were modified with frequencies of 0.1.2–4.67% (Figure 5A). However, the frequencies of the targets did not show any obvious correlation with chromosome position or with distance from the origin of replication (Ori) or the terminus (Term; Supplementary Figure S5), suggesting that the SMGE method does not display target bias and is of considerable importance for achieving multiplex genome engineering. Similarly, allelic replacement frequencies of hierarchical CAGE also do not correlate with any factors (Isaacs et al., 2011). Notably, simultaneous editing of up to 4, 6, and 8 genes was separately achieved using three different pMGRn vectors (Figure 5B), suggesting that the number of simultaneously modified genes in the chromosome could increase with the number of target genes in the integration plasmid. Taken together, the multiplex genome engineering method developed in this study is time-saving and affordable owing to the one-step transformation of an all-in-one plasmid compared to the previous methods, which always require multi-round transformations or multi-step modifications in a stepwise manner (Isaacs et al., 2011; Lajoie et al., 2013; Dalia et al., 2014).

The selectable markers and long homologous arms of genetically modified genes on the integration plasmid are of great significance to achieve simultaneously multiplex engineering by the SMGE method. To screen multiple mutants with a simple and fast protocol, the editing frequencies relying on selectable markers are relatively low, which could be improved by optimizing the recombination machinery. Moreover, the construction of the integration plasmid is increasingly difficult as the number of modified genes increases. Therefore, the SMGE method might be limited by the number of targeted genes in the all-in-one plasmid. However, it could be improved by the rapid advances of multiple/large DNA assembly and synthetic technology (Smanski et al., 2014; Yan et al., 2018). To our knowledge, about 4 weeks are required for the generation of large DNA fragments (>15 kb), including synthesis of all DNA fragments, one-step assembly, ligation, transformation, screening, and sequencing. The time required and the available technologies can be obtained from the GenScript Company’s Web site.^1^ In our study, all DNA fragments were PCR amplified within 1–2 days, and the time for insertion of a large DNA fragment into the plasmid was reduced to 1–2 weeks.

Aromatic amino acids and their intermediates have been used as important precursors for the production of diverse aromatic acids, hydrocarbons, ketones, alcohols, and aromatic natural compounds (Huccetogullari et al., 2019). To improve tyrosine production in natural microbes, several metabolic engineering strategies have been developed by enhancing precursors, releasing feedback inhibition, blocking branch pathways, and optimizing the transport systems in *E. coli* and *Corynebacterium glutamicum* (Huccetogullari et al., 2019). To demonstrate the application of the SMGE method, this study first globally optimized the tyrosine biosynthesis pathway of *B. subtilis* (Figure 6A). The yield-improved variants were rapidly selected from the mutant pool using high-throughput screening in 48-well plates (Figure 6B). Different combinations of genetic modifications in the tyrosine synthesis pathway had been assessed in various variants, suggesting that targets *aroA*, *pheA*, and *csrA* could significantly increase tyrosine production (Figure 6C). In previous studies, the tyrosine production has been increased through overexpression of *ppsA*, *tktA*, and *aroG* genes and point mutation of *aroG*, *AroF*, and *tyrA* in *E. coli* (Kim et al., 2015, 2018; Cui et al., 2019). In this study, *eno* overexpression and *pyk* deletion could enhance tyrosine production in *B. subtilis*, but these mutants would remarkably reduce cell growth and could not produce the highest tyrosine yield (Figure 6C). Besides, the CsrA is an RNA-binding protein that participates in regulation at the overall metabolic level (Berndt et al., 2019). It positively regulates the pyruvate kinase PyK responsible for the decomposition of PEP and negatively regulates the PEP carboxykinase PckA and synthase PpsA responsible for the synthesis of PEP. The inactivation of CsrA can relieve its regulation of these enzymes and thereby enhance the PEP and tyrosine synthesis in *B. subtilis*.

Among aromatic natural compounds, resveratrol (3, 5, 4-trihydroxystilbene), a well-known pharmacological polyphenol in humans, is produced as a secondary metabolite by plants. Owing to its limited production and difficulty in purification from natural hosts, numerous studies have advances in improving resveratrol biosynthesis in microbial hosts, which grow rapidly and can be easily genetically manipulated (Tatsis and O’Connor, 2016; Thapa et al., 2019). However, the development of efficient and feasible microbial fermentations of resveratrol from glucose remains very challenging. Through introducing the heterologous pathway genes from the plants, microbial production of resveratrol has been achieved by the addition of the precursor tyrosine, phenylalanine, or *p*-coumaric acid (Wu et al., 2013; Thapa et al., 2019). So far, the available studies indicate that the amount of precursor is one of the key factors to increase resveratrol production. To efficiently synthesize resveratrol from glucose, metabolic engineering has demonstrated its superiority to increase the pool of precursors and maintain cell physiochemical conditions for the production of resveratrol (Huccetogullari et al., 2019). In this study, the codon-optimized heterologous genes, *tal*, *4 cl*, and *sts* separately from *Herpetosiphon aurantiacus*, *Arabidopsis thaliana*, and *Vitis vinifera*, were expressed in the optimized variant to produce resveratrol of 2.11 ± 0.08 mg l^−1^ (Figure 6D). However, the WT strain harboring these genes could not produce measurable resveratrol from the basal tyrosine pool. The reason might be that strictly metabolic inhibition of tyrosine synthesis could maintain a low concentration of tyrosine for cell growth, which is not sufficient for multiple steps of enzymatic catalysis to produce detectable resveratrol. To our knowledge, the overall production of tyrosine and resveratrol still does not meet the demand of commercial production (Thapa et al., 2019). *B. subtilis* can be used as a new chassis strain for the optimization of tyrosine and resveratrol production in the future. Therefore, the SMGE method developed here can be optimized for biosynthetic pathways for producing commercially important biochemical products from the genetically diverse mutant pool in a considerably short timescale.

Taken together, a new multiplex genome engineering method was developed using one-step natural transformation of an all-in-one plasmid by combining competency factors and recombinase. Multiplex genetically diverse mutant pools were efficiently generated using SMGE in a considerably short timescale, which will be advantageous for the selection of overproducing strains *via* a high-throughput 96-well/48-well approach. In addition, this method can edit all genomic loci and tandem genetic modifications (deletions, replacements, insertions, and point mutations) in a simple and easy operation. Therefore, the SMGE method can be used to generate a highly efficient, affordable, and versatile platform for large-scale genetic engineering and biotechnology applications.

## Data Availability Statement

The original contributions presented in the study are included in the article/Supplementary Material, and further inquiries can be directed to the corresponding authors.

## Author Contributions

TWe and AD designed and supervised the study. AD and ZS performed the experiments and all data analysis. TWa, DC, LL, FH, and SW performed the selection of the variant pools and fermentation experiments. AD wrote the manuscript with the revision from TWe and SW. All of the authors have approved the final paper.

## Conflict of Interest

Author Fei Huang is employed by Zenbio Biotech Co., Ltd. (Chengdu, China).

The remaining authors declare that the research was conducted in the absence of any commercial or financial relationships that could be construed as a potential conflict of interest.

## Publisher’s Note

All claims expressed in this article are solely those of the authors and do not necessarily represent those of their affiliated organizations, or those of the publisher, the editors and the reviewers. Any product that may be evaluated in this article, or claim that may be made by its manufacturer, is not guaranteed or endorsed by the publisher.

## Footnotes

^1^ https://www.genscript.com/synthetic-biology-gene-synthesis-service.html
