# Supplementary material for: Simultaneous Multiplex Genome Engineering via Accelerated Natural Transformation in Bacillus subtilis
Source: Front Microbiol. 2021 Aug 17;12:714449. doi: 10.3389/fmicb.2021.714449 (PMC8416114; doi:10.3389/fmicb.2021.714449)
Supplement: Supplementary file 2 [file Data_Sheet_1.PDF]

Supplementary Materials

Simultaneous Multiplex Genome Engineering via Accelerated Natural Transformation in *Bacillus subtilis*

Aihua Deng[1], Zhaopeng Sun[1], Tiantian Wang[1,2], Di Cui[1], Lai Li[1,2], Shuwen Liu[1,3] , Fei Huang[4],Tingyi Wen[1,3,5,*]

[1]CAS Key Laboratory of Pathogenic Microbiology and Immunology, Institute of Microbiology, Chinese Academy of Sciences, Beijing 100101, China

[2]University of Chinese Academy of Sciences, Beijing 100049, China

[3]China Innovation Academy for Green Manufacture, Chinese Academy of Sciences, Beijing, 100049, China

[4]Zenbio Biotech Co., Ltd. (Chengdu, China)

[5]Savaid medical school, University of Chinese Academy of Sciences, Beijing, 100049, China

*Correspondence to: wenty@im.ac.cn

METHODS

Construction of Recombinant and Expression Plasmids

For the construction of pMGRn plasmids, upstream and downstream homologous arms of each target (the knockout/ inserted/ mutated fragment) were PCR-amplified from the chromosomal DNA of *Bacillus subtilis* W168 using primers listed in Table S2. The *kan*^{R}, *erm*^{R}, and *spec*^{R} genes were amplified using plasmids pDG780, pDG641, and pDG1727 as templates, respectively (GueroutFleury et al., 1995). The homologous arms and integrated genes of multiple target genes were self-assembled using the Gibson assembly method, generating recombination vectors pMGRn (Gibson et al., 2009). The DNA sequences for all plasmids constructed in this study were shown in the Supplementary sequences.

To construct the recombinant plasmid pMGR2 for simultaneous deletion of two genes *upp* and *divIVA*, the resistant genes, upstream and downstream homologous sequences were amplified using primers P1 to P12 (Table S2). The upstream and downstream DNA fragments flanking the gene *divIVA* were amplified using W168 chromosome as the template and jointed with the resistant gene *Erm*^{R} by Splice Overlap Extension (SOE) PCR. The fused flank homologous sequence was digested with *Sph*I and ligated with pMD19 and pWYE799 (Sun et al., 2015) treated with the same restriction endonuclease to generate plasmids pWYE818 (T-G2) and pWYE819 (T-G1-G2, designated as pMGR2), respectively (Table S1). The fragment G1 was PCR amplified by the primers P9 and P10 using pWYE799 (T-G1) as the template. The fragment G2 was PCR amplified by the primers P1 and P6 using pWYE818 (T-G2) as the template. The all-in-one PCR product for two target genes (G1-G2) was PCR amplified by the primers P1 and P10 using pWYE819 (T-G1-G2) as the template. The G1 chromosome (G1 chr) was extracted from *B. subtilis* BS069 (Sun et al., 2015). The G2 chromosome (G2 chr) was extracted from the mutant BS153 (W168 ⊿*divIVA:: erm*^{R}), which was obtained by the transformation of the plasmid pWYE818 into the W168 strain to delete *divIVA* and integrate *erm*^{R} (Table S1).

To construct the recombinant plasmid pMGR6, upstream and downstream homologous of mutant *upp**, *ropB**, *amyE**, *purA**, and *thrC** were amplified using W168 chromosome as the template (see Table S2 for the detailed mutant sites and primes P13-P40). The upstream and downstream DNA fragments flanking 5 target genes were jointed by SOE-PCR and ligated into the pMD19-T vector (Takara, Dalian, China) to generate pMD19-T-HA-*upp*-ropB*-amyE*-purA*-thrC**. The plasmid pWYE753 (Li et al., 2011) was digested with *Bam*HI and *Kpn*I to obtain the DNA fragment *deoDU- kan^{R}-deoDD* containing homologous fragments of *deoD* flanking *kan^{R}*. The purified DNA fragment was then ligated with pMD19-T-HA-*upp*-ropB*-amyE*-purA*-thrC** treated with the same endonucleases to generate pWYE792 (designated as pMGR6).

To overexpress the recombinase in *B. subtilis*, the primers P93 and P94 were used to PCR amplify the gene *gp35* using the chromosome of *B. subtilis* BS045 as the template (Sun et al., 2015). After digested with *Bam*HI and *Xma*I, the purified PCR fragment was ligated with pHCMC04 treated with the same endonucleases to generate pWYE895 (Table S1). To overexpress the competent factor in *B. subtilis*, the fragment *PmtlA-comKS* was amplified by SOE-PCR using the W168 chromosome as the template (see Table S2 for detailed primes P85-P92). The purified fragment was then ligated in the *Kpn*I site of pHCOMC04 and pWYE895 to generate pWYE896 and pWYE897 (Table S1). The plasmids pWYE895, pWYE896, and pWYE897 were further transformed into *B. subtilis* W168 to obtain strains WYB106 (W168/pHCMC04-*gp35*), WYB107 (W168/pHCMC04-*comKS*), and WYB108 (W168/pHCMC04-*gp35-comKS*), respectively (Tables S1).

To construct the recombinant plasmid pMGR10, primers P1 and P6 were used to amplify the DNA fragment *divIVAU-erm^{R}-divIVAD* using pWYE818 as the template. After digestion with *Sph*I, the purified fragment *divIVAU-erm^{R}-divIVAD* was ligated with pWYE792 to obtain pWYE792-*divIVAU-erm^{R}-divIVAD*. The upstream and downstream homologous sequences of mutant *recU**, *teL**, and *trpC* *were amplified

using W168 chromosome as the template (see Table S2 for detailed mutation sites and primes P41-P54). The upstream and downstream DNA fragments flanking 3 target genes were jointed by SOE-PCR and ligated into the *Bam*HI and *Xba*I sites of pWYE792-*divIVAU-erm*^{R}-*divIVAD* to generate pWYE830 (designated as pMGR10).

To construct the recombinant plasmid pMGR15, the upstream and downstream homologous sequences of targeted genes *hisD*, *metA*, *lysA*, *ilvA*, and *aprE* were PCR amplified using 168 chromosome as the template (detailed primes P55-P84 and mutation sites in the Table S2). The upstream and downstream DNA fragments flanking 5 target genes were jointed by SOE-PCR and ligated into the *Bam*HI sites of pWYE830 to generate pWYE944 (designated as pMGR15).

To construct the recombinant plasmid pMGR-tyr10, the upstream and downstream homologous sequences of targeted genes *tkt*, *eno*, *pyk*, *aroA*, *aroF*, *trpE*, *tyrA*, *pheA*, and *csrA* were PCR amplified using W168 chromosome as the template (see Table S2 for detailed mutation sites and primes P97-P158). Specifically, the upstream and downstream DNA fragments flanking *trpE* were jointed with *erm*^{R} by SOE-PCR to obtain the fused fragment *trpEU-erm*^{R}-*trpED*. The upstream and downstream DNA fragments flanking *pyK* were jointed with *eno* and *kan*^{R} by SOE-PCR to obtain the fused fragment *pykU-eno-kan*^{R}-*pykD*. The upstream and downstream DNA fragments flanking *aroA* were jointed by SOE-PCR to obtain the mutant *aroA*$^{C575T/G576}$. The upstream and downstream DNA fragments flanking *pheA* were jointed with *spec*^{R}, P_{spovG}, and *aroA*$^{C575T/G576}$ by SOE-PCR to obtain the fused fragment *pheU-spec*^{R}-P_{spovG}-*aroA*$^{C575T/G576}$-*pheD*. The upstream and downstream DNA fragments flanking *upp* were jointed with *tkt* by SOE-PCR to obtain the fused fragment *uppU-tkt-uppD*. The upstream and downstream DNA fragments flanking *aroH* were jointed by SOE-PCR to obtain the mutant *aroH*$^{T261A/G262A/C263A /A268T/G269C}$. The upstream and downstream DNA fragments flanking *tyrA* were jointed by SOE-PCR to obtain the mutant *tyrA*$^{T937G/C939G}$. The upstream and downstream DNA fragments flanking *csrA* were jointed by SOE-PCR to obtain the mutant *csrA*$^{1\text{-}162}$. The

fused fragments of *trpEU-erm*^{R}*-trpED, csrA*$^{1\text{-}162}$*, pheU-spec*^{R}*-P*$_{spovG}$*-aroA*$^{C575T/G576}$*-pheD,*

aroH$^{T261A/G262A/C263A\,/A268T/G269C}$, *tyrA*$^{T937G/C939G}$, and *aroA*$^{C575T/G576}$ were first assembled into the T vector to obtain pMGR-tyr8 by the Genscript Company. Then, the fused fragments of *uppU-tkt-uppD* and *pykU-eno-kan*^{R}*-pykD* were jointed by SOE-PCR and ligated into the pMGR-tyr8 to generate pWYE995 (designated as pMGR-tyr10).

To construct genetic manipulating vectors for scarless knocking resistant genes, the upstream and downstream homologous sequences of targeted genes *spec*^{R}, *erm*^{R}, and *kan*^{R} were PCR amplified using mutants as the template (see Table S2 for detailed primes P159-P172). The upstream and downstream DNA fragments flanking of each target gene were jointed by SOE-PCR and ligated into the *Kpn*I site of pWYE486 (Wu et al., 2018) to generate pWYE900, pWYE901, and pWYE904.

To express the heterologous genes for resveratrol synthesis, the codon optimization of genes *tal*, *4cl,* and *sts* separately from *Herpetosiphon aurantiacus*, *Arabidopsis thaliana,* and *Vitis vinifera* were performed by the Genscript Company. According to thecodon preference of *B. subtilis* (http://www.jcat.de/), three fragments of *tal*^{BS}, *P*$_{veg}$*-4cl*^{BS}, and *P*$_{43}$*-sts*^{BS} were synthesized for the construction of the expression plasmid. To eliminate a *Bgl*II site and a *Pst*I site of pHCMC04, primers P173 and P174 were used for PCR amplification of pHCMC04. The fragment was digested with *Dpn*I and transformed in the host cell to obtain pHCMC04*. Primers P175 and P176 were used to amplify the optimized *tal* gene, which was ligated into the *Bam*HI site of pHCMC04* to obtain pHCMC04*-*tal*^{BS}. Then, primers P176 and P177 were used to amplify the optimized fragment of *P*$_{veg}$*-4cl*^{BS}, which was ligated into the *Bam*HI site of pHCMC05-*tal*^{BS} to obtain pHCMC04*- *tal*^{BS}*-P*$_{veg}$*-4cl*^{BS}. Finally, primers P178 and P179 were used to amplify the optimized fragment of *P*$_{43}$*-sts*^{BS}, which was ligated into the *Bgl*II site of pHCMC04*- *tal*^{BS}*-P*$_{veg}$*-4cl*^{BS} to obtain pWYE898 (pHCMC04*-*tal*^{BS}*-P*$_{veg}$*-4cl*^{BS}*- P*$_{43}$*-sts*^{BS}). The plasmid pWYE898 was further transformed in the variant Type 2 and original W168 (WT) to obtain strains BS191 and BS192 (Tables S1).

Table S1. Bacterial strains and plasmids used in this study.

Strains/ plasmids	Relevant characteristics	References/ sources
**Strains**		
E. coli EC135	General cloning host strain, TOP10 *Δdcm::FRT recA*$^{+}$*Δdam::FRT, genotype of R-M systems: mcrA Δ(mrr-hsdRMS-mcrBC) Δdcm::FRT Δdam::FRT*	(Zhang et al., 2012)
B. subtilis W168	Prototroph, *rpoB*18	BGSC
B. subtilis BS056	W168*ΔlacA*::P_{xylA}-*beta-erm*^{R}	(Sun et al., 2015)
B. subtilis BS060	W168*ΔlacA*::P_{xylA}-*gp35-erm*^{R}	(Sun et al., 2015)
B. subtilis BS061	W168*ΔlacA*::P_{xylA}-*orf48-erm*^{R}	(Sun et al., 2015)
B. subtilis WYB106	W168/pHCMC04-*gp35*	This study
B. subtilis WYB107	W168/pHCMC04-*comKS*	This study
B. subtilis WYB108	W168/pHCMC04 -*gp35-comKS*	This study
B. subtilis BS153	W168 *Δ divIVA:: erm*^{R}	This study
B. subtilis BS155	WYB108 *Δ deoD::kan*^{R} *Δ rpoB amyE* thrC**	This study
B. subtilis BS156	WYB108 *Δ divIVA::erm*^{R} *Δ deoD::kan*^{R} *rpoB* amyE* thrC* tetL**	This study
B. subtilis BS157	WYB108 *Δ divIVA::erm*^{R} *Δ deoD::kan*^{R} *rpoB* amyE* trpC* thrC**	This study
B. subtilis BS171	WYB108 *Δ divIVA::erm*^{R} *Δ deoD::kan*^{R} *rpoB* amyE* upp* tetL* thrC* trpC**	This study

B. subtilis BS182	W168 *Δpyk::eno*	This study
B. subtilis BS183	W168*ΔcrsA ΔpheA::aroA**	This study
B. subtilis BS184	W168 *tyrA*ΔcrsAΔpheA::aroA**	This study
B. subtilis BS185	W168 *Δpyk::eno Δupp::tkt ΔpheA::aroA**	This study
B. subtilis BS186	W168 *aroA* Δpyk::eno ΔpheA::aroA**	This study
B. subtilis BS187	W168 *tyrA* Δpyk::eno ΔpheA::aroA**	This study
B. subtilis BS188	W168 *ΔcrsA Δpyk::eno ΔpheA::aroA**	This study
B. subtilis BS189	W168 *tyrA* ΔtrpE ΔpheA::aroA**	This study
B. subtilis BS190	W168 *tyrA* Δpyk::eno Δupp::tkt ΔpheA::aroA**	This study
B. subtilis BS191	W168/pWYE898	This study
B. subtilis BS192	BS183/ pWYE898	This study
**Plasmids**		
pHCMC04	*E. coli-Bacillus* shuttle plasmid, theta replicative, carrying P_{xyl} promoter, *amp*^{R}, and *cm*^{R}	BGSC
pMD19-T	*E. coli* cloning T vector, oriC replicative, carrying *amp*^{R}	Takara, Dalian, China
pWYE486	Genetic manipulating vector for scarless gene editing in the *B. subtilis*, carrying oriC replicative, P_{xyl} promoter, *amp*^{R}, and *erm*^{R}	(Wu et al., 2018)
pWYE753	Integrative vector for deleting *deoD* in W168, conferring *kan*^{R}	(Li et al., 2011)

pWYE792	Integrative vector pMGR6 derived from T vector for editing six genes of W168	This study
pWYE799	Integrative vector derived from T vector for deleting *upp* of W168, conferring kan^R	(Sun et al., 2015)
pWYE818	Integrative vector derived from T vector for deleting *divIVA* of W168, conferring erm^R	This study
pWYE819	Integrative vector pMGR2 derived from T vector for deleting *upp* and *divIVA* of W168, conferring kan^R and erm^R	This study
pWYE830	Integrative vector pMGR10 ((T-HA-*deoD-recU-tetL-purA- trpC-upp- thrC- ropB-amyE-dilvA*) for editing ten genes from W168	This study
pWYE895	Expression vector harboring *gp35*	This study
pWYE896	Expression vector harboring *comKS*	This study
pWYE897	Expression vector harboring *gp35* and *comKS*	This study
pWYE898	Expression vector harboring *tal*, *4cl*, and *sts*	This study
pWYE900	Genetic manipulating vector for scarless knocking $spec^R$ gene	This study
pWYE901	Genetic manipulating vector for scarless knocking erm^R gene	This study
pWYE902	Genetic manipulating vector for scarless knocking kan^R gene	This study
pWYE944	Integrative vector pMGR15 (T-HA-*ropB-amyE-aprE-dilvA-deoD-ilvA-metA-recU-trpC -lysA-thrC-hisD-upp-purA-tetL*) for editing fifteen	This study

	genes in *B. subtilis*	
pWYE995	Integrative vector pMGR-tyr10 (T-HA *tyrA**-*aroH**-*aroA**- *pheA*-*aroA*- *trpE*- *csrA*- *tkt*- *pyk*-*eno*) for optimization of the tyrosine biosynthesis pathway in *B. subtilis*	This study

kan^R, kanamycin resistance; *cm*^{R}, chloramphenicol resistance; *erm*^{R}, erythromycin resistance; *spec*^{R}, spectamycin resistance; *rif*^{R}, rifampicin resistance; *, point mutation.

Table S2. PCR primers used in this study.

Primers	Sequence（5'-to-3'）	Description
P1	ACATGCATGCTCGTGTTTTCTGAGACAG	Forward primer for amplification of upstream homologous sequence for *divIVA* deletion
P2	ATATTTTTGTTCATGATGCCACCTCCAT	Reverse primer for amplification of upstream homologous sequence for *divIVA* deletion
P3	ATGG AGGTGGCATCATGAACAAAA ATAT	Forward primer for amplification of *erm*^{R} gene
P4	GATAATCAGAGAATTTATTTCCTCCCGT	Reverse primer for amplification of *erm*^{R} gene
P5	ACGGGA GGAAATAAATTCTCTGAT TATC	Forward primer for amplification of downstream homologous sequence for *divIVA* deletion
P6	ACATGCATGCATGTAAAACAAATTTCGGGC	Reverse primer for amplification of downstream homologous sequence for *divIVA* deletion
P7	GCGGCAAAAATTCGGTGACA	Forward primer for identification of *divIVA*
P8	TTTGCATACGGAGGTCCGTC	Reverse primer for identification of *divIVA*
P9	CGGGATCCAACAGTTCCC AGAATAAGGC	Forward primer used for cloning the *upp*-*kan*^{R} cassette using the genome DNA of *B. subtilis* BS069 as the template (Sun et al., 2015)
P10	CGGGATCCTCCTCGTACAATCGACTTTAAG	Reverse primer used for cloning the *upp*-*kan*^{R} cassette using the genome DNA of *B. subtilis* BS069 as the template (Sun et al., 2015)
P11	CGTGAAGAGTTTGGCAAGAA	Forward primer for identification of *upp*
P12	ATTCTTCTGACGAGCAGTAA	Reverse primer for identification of *upp*
P13	GCTACACTCATGGCATTTGA	Forward primer for amplification of upstream homologous sequence for mutation *upp*^{C309G}
P14	TTCTGGATCACGCTAAAGGCCGACATGT	Reverse primer for amplification of upstream homologous sequence for mutation *upp*^{C309G}
P15	ACATGTCGGCCTTTAGCGTGATCCAGAA	Forward primer for amplification of downstream homologous sequence for mutation *upp*^{C309G}
P16	GAATGCTCGTCTTGAAATTCTTCCACACCC	Reverse primer for amplification of downstream homologous sequence for mutation *upp*^{C309G}
P17	GGGTGTGGAAGAATTTCAAGACGAGCATTC	Forward primer for amplification of upstream homologous sequence for mutation *rpoB*^{A1445G}

P18	GCTGAATTAACGCGCAAGCGTCGTCTGTCA	Reverse primer for amplification of upstream homologous sequence for mutation *rpoB*^{A1445G}
P19	TGACAGACGACGCTTGCGCGTTAATTCAGC	Forward primer for amplification of downstream homologous sequence for mutation *rpoB*^{A1445G}
P20	CTGCATATGCGAATTTTTCTTCAATCAGCT	Reverse primer for amplification of downstream homologous sequence for mutation *rpoB*^{A1445G}
P21	TACTTAGAAAACGGAATCGGT	Forward primer for identification of *rpoB*
P22	TCCAACAAATGGCGCTTCCGGCT	Reverse primer for identification of *rpoB*
P23	AGCTGATTGAAGAAAAATTCGCATATGCAG	Forward primer for amplification of upstream homologous sequence for mutation *amyE*^{G490T}
P24	TGGCAACCGTTACTAAGGTACTGAACAAG	Reverse primer for amplification of upstream homologous sequence for mutation *amyE*^{G490T}
P25	CTTGTTCAGTACCTTAGTAACGGTTGCCA	Forward primer for amplification of downstream homologous sequence for mutation *amyE*^{G490T}
P26	TATTAAAGTTCATAAACACAATATGAAG	Reverse primer for amplification of downstream homologous sequence for mutation *amyE*^{G490T}
P27	AAGGAGGAAGCGGAAGAATGAAGTAAGAGG	Forward primer for identification of *amyE*
P28	TGCGACTCTACCCATGTCACTAGCTTGTC	Reverse primer for identification of *amyE*
P29	GACTGGCTTTTATAAATGTCTTCAGTAGTT	Forward primer for amplification of upstream homologous sequence for mutation *purA*^{T324A}
P30	CCAATTTCAAATGTTACGGCAGAATGACG	Reverse primer for amplification of upstream homologous sequence for mutation *purA*^{T324A}
P31	CGTCATTCTGCCGTAACATTTGAAATTGG	Forward primer for amplification of downstream homologous sequence for mutation *purA*^{T324A}
P32	ATGTAAAGTTAGCGCAGCATCGTTTAAGAC	Reverse primer for amplification of downstream homologous sequence for mutation *purA*^{T324A}
P33	GTTAACGGAGGTGCACGGACAT	Forward primer for identification of *purA*
P34	GGCTTGCAGACATATTCTATT	Reverse primer for identification of *purA*
P35	GTCTTAAACGATGCTGCGCTAACTTTACAT	Forward primer for amplification of upstream homologous sequence for mutation *thrC*^{C363A}
P36	ATAATCTCGGCTCCTTACATGACAGCTTGA	Reverse primer for amplification of upstream homologous sequence for mutation *thrC*^{C363A}
P37	TCAAGCTGTCATGTAAGGAGCCGAGATTAT	Forward primer for amplification of downstream homologous sequence for mutation *thrC*^{C363A}
P38	TGCCGCCGCACCCTCAGCTTC	Reverse primer for amplification of downstream homologous sequence for mutation *thrC*^{C363A}
P39	GAAGGGAACGGTTGGAGCTAAT	Forward primer for identification of *thrC*
P40	GAACAGCATGTCGGCTTCGTTCAT	Reverse primer for identification of *thrC*
P41	CGGGATCCAAGAAGCCAAAAGCGACC	Forward primer for amplification of *recU* ^{A107C} using pWYE846 as the template (Wu et al., 2018)
P42	ACCCTGTGGACTGGTACAAAACAAATACCG	Reverse primer for amplification of *recU* ^{A107C}
P43	GTGGAAGTAACCCAATCT	Forward primer for amplification of upstream homologous sequence for mutation *tetL*^{T301C}

P44	TGATATGATCTTATCATATATAGACTAAGG	Reverse primer for amplification of upstream homologous sequence for mutation *tetL*^{T301C}
P45	CCTTA GTCTATATATGATAAGATCATATCA	Forward primer for amplification of downstream homologous sequence for mutation *tetL*^{T301C}
P46	TAATCCCTGCCATATCAA	Reverse primer for amplification of downstream homologous sequence for mutation *tetL*^{T301C}
P47	CGGTATTTGTTTTGTACCAGTCCACAGGGT	Forward primer for amplification of *tetL*
P48	GATGTTTTCAAGCATTGTATTCACCCAGTT	Reverse primer for amplification of *tetL*
P49	ATGCTTGAAA ACATCATTAA AC	Forward primer for amplification of upstream homologous sequence for mutation *trpC*^{C302A}
P50	CTGAGTACAGGTATCTAAACAGCGCGCTTTA	Reverse primer for amplification of upstream homologous sequence for mutation *trpC*^{C302A}
P51	TAAAGCGCGCTGTTTAGATACCTGTACTCAG	Forward primer for amplification of downstream homologous sequence for mutation *trpC*^{C302A}
P52	TCACTCCCCAAACAAAGCATGG	Reverse primer for amplification of downstream homologous sequence for mutation *trpC*^{C302A}
P53	AACTGGGTGAATACAATGCTTGAAAACATC	Forward primer for identification of *trpC*
P54	GCTCTAGATCACTCCCCAAACAAAGCATG	Reverse primer for identification of *trpC*
P55	ACTGGATCCATTAAGCTCAACGGTTCAATTGA	Forward primer for amplification of upstream homologous sequence for *hisD* deletion
P56	TTGATCTGGCATTTCCGGCATCGATGGAACGCT TGA	Reverse primer for amplification of upstream homologous sequence for *hisD* deletion
P57	ATCGATGCCGGAAATGCCAGATCAATTGAAGC GAGA	Forward primer for amplification of downstream homologous sequence for *hisD* deletion
P58	ATACATATCAGCATATCGCCCAACGCTTCGAGC AGC	Reverse primer for amplification of downstream homologous sequence for *hisD* deletion
P59	AATCTTCGGTTTATATTAGCGAAGCCGAT	Forward primer for identification of *hisD*
P60	TTCAATCATGTGGTGGGTATTTGT	Reverse primer for identification of *hisD*
P61	ACATGATTGAAATGTAAACATCAATTAAGAACT	Forward primer for amplification of upstream homologous sequence for *metA* deletion
P62	AGTTTCTTGGTATAATAGGCAACGTGCCACCTC CATTA	Reverse primer for amplification of upstream homologous sequence for *metA* deletion
P63	ACGTTGCCTATTATACCAAGAAACTCCTTATGA ATGGGA	Forward primer for amplification of downstream homologous sequence for *metA* deletion
P64	TTAAACCCATTGCTGAAATTAATACTCTTTTATT GGTA	Reverse primer for amplification of downstream homologous sequence for *metA* deletion
P65	TGCTGATATGTATCATATAATT	Forward primer for identification of *metA*
P66	ACGATAAAACTGTTTCCCGATTGAGA	Reverse primer for identification of *metA*
P67	AACAGTTTTATCGTGGATTTACAGTGCTAATTAT TGCCA	Forward primer for amplification of upstream homologous sequence for *lysA* deletion
P68	TTGTATCTATATTCTGTCATTCCCTCTTTCTCCGC	Reverse primer for amplification of upstream

	TTAT	homologous sequence for *lysA* deletion
P69	AGAGGGAATGACAGAATATAGATACAATCGCATTCCAGAT	Forward primer for amplification of downstream homologous sequence for *lysA* deletion
P70	TTATTCATGAGCTTTGATAATAAAGCTGACATCGAA	Reverse primer for amplification of downstream homologous sequence for *lysA* deletion
P71	CAGCAATGGGTTTAATTGCCGCTGTT	Forward primer for identification of *lysA*
P72	GAAGTATCCGGGACTGCCGCTTTAACT	Reverse primer for identification of *lysA*
P73	AGTCCCGGATACTTCTCTCCGATTAGATGTTGAAATGTA	Forward primer for amplification of upstream homologous sequence for *ilvA* deletion
P74	TTGCGCGGTTGTGTAGATTCCTTTCTTGTTTTAAATCCCTA	Reverse primer for amplification of upstream homologous sequence for *ilvA* deletion
P75	AAGAAAGGAATCTACACAACCGCGCAAAATGACAGA	Forward primer for amplification of downstream homologous sequence for *ilvA* deletion
P76	TAACCAATATCGGAGCCTGATAATCTGCCCACTTCATC	Reverse primer for amplification of downstream homologous sequence for *ilvA* deletion
P77	AAGCTCATGAATAAGGGTTTCTTC	Forward primer for identification of *ilvA*
P78	CCTTGATAACCTTCATGACATCCAT	Reverse primer for identification of *ilvA*
P79	AAGGTTATCAAGGACGAATTTTTTCGACAGGAATT	Forward primer for amplification of upstream homologous sequence for *aprE* deletion
P80	AACCTGCTTCTTTGCTTCTCACTCTTTACCCTCTCCTT	Reverse primer for amplification of upstream homologous sequence for *aprE* deletion
P81	AAGAGTGAGAAGCAAAGAAGCAGGTTCCTCCATACCTGCTT	Forward primer for amplification of downstream homologous sequence for *aprE* deletion
P82	ATCGGATCCTGTTTTGAGCTTTGCGGTTACGCCT	Reverse primer for amplification of downstream homologous sequence for *aprE* deletion
P83	CCGATATTGGTTAAACAGCGGCGCAA	Forward primer for identification of *aprE*
P84	CACGAAATGGGCCATTATGTCATGAA	Reverse primer for identification of *aprE*
P85	TAATTGTCACAGTCATGTGCCAAAGTCCTCTTTACTTTCAATTGTATAGGGACTGTAAG	Primer 1 for amplification of the *PmtlA*
P86	AGGGACTGTAAGCGTTTTAACATAGAGTCAAAGGGAAGCATCATTCTCTGAACATATTT	Primer 2 for amplification of the *PmtlA*
P87	TTCTCTGAACATATTTCTTAAGTGCAGAAAAAGGATGGAGG	Forward primer for amplification of *comK*
P88	TTTCGTTCTCTTCCAAATACACCTATTTTTCTAATACCGTTC	Reverse primer for amplification of *comK*
P89	GAACGGTATTAGAAAAATAGGTGTATTTGGAAGAGAACGAAA	Forward primer for amplification of *comS*
P90	AACATCACTGCGTACTGGAAATAATAAAAAAGCCGGATTAATAATCTGGCTTTTTATAT	Reverse primer for amplification of *comS*
P91	TCGATTTGTTTTTCCAGATCTCGAGGCCTGCACTTTTTATTTTTAAAAAATTGTCACA	Forward primer for amplification of *PmtlA-comKS*

P92	AGCTATGACCATGATTACGCCAAGCTTGCATGCCAAATAATAAAAAAGCCGGATTAATA	Reverse primer for amplification of *PmtlA-comKS*
P93	TCTTGTTTTTAGTTGCCATAGGATCCCATTTCCCCCTTTGATTTTTAGATATCACT	Forward primer for amplification of *gp35*
P94	TCATTAGGCGGGCTGCCCCGGGGACGTCGACTCTAGCTATTCATTTGTTTCCCCTCCTA	Reverse primer for amplification of *gp35*
P95	TAACTTTAACAACTCGATTTGTTTTTCCAGATCCTAACTTATAGGGGTAACACTTAA	Forward primer for identification of *gp35*
P96	ACAGCTATGACCATGATTACGCCAAGCTTGCATGCCTATTCATTTGTTTCCCCTCCTAA	Reverse primer for identification of *gp35*
P97	ACCGCAAAAGGGCAGAACAATGCATTGAAGCATGTACTTGATGAAATTGGTAT	Forward primer for amplification of upstream homologous sequence for *tkt* integration
P98	AACAGATTTTTTTTCGATTGTATCCATACTGTGTTTCAGCTCCTTTTTTATTG	Reverse primer for amplification of upstream homologous sequence for *tkt* integration
P99	CAATAAAAAAGGAGCTGAAACACAGTATGGATACAATCGAAAAAAAATCTGTT	Forward primer for amplification of *tkt* gene
P100	GAAACCCCCTTTTGGGGATTTCATTTTTATTTGTTGATAAGAGCTTTAACACGGTT	Reverse primer for amplification of *tkt* gene
P101	AACCGTGTTAAAGCTCTTATCAACAAATAAAAAATGAAATCCCCAAAAGGGGGTTTC	Forward primer for amplification of downstream homologous sequence for *tkt* integration
P102	ACCGGCCCACAATGCGATATCGCCGGCATGAAAAATAATGTATAGCTTGGATACGGCAA	Reverse primer for amplification of downstream homologous sequence for *tkt* integration
P103	ACTGACTGGTAAGGTTGCGG	Forward primer for identification of *tkt* integration
P104	ATTATCTGTCTCCTGATGAA	Reverse primer for identification of *tkt* integration
P105	TTGCCGTATCCAAGCTATACATTATTTTTCATGCCGGCGATATCGCATTGTGGGCCGGT	Forward primer for amplification of upstream homologous sequence for *pyk* deletion
P106	ACGAGCGTAAACATCAACGATGTAAGGCATTTGGTTCACTTCCTTCTGAAATCTTCA	Reverse primer for amplification of upstream homologous sequence for *pyk* deletion
P107	TGAAGATTTCAGAAGGAAGTGAACCAAATGCCTTACATCGTTGATGTTTACGCTCGT	Forward primer for amplification of *eno* gene
P108	TTCGATTGTGAATAGGATGTATTCATCTATTATTTGTTAAGGTTGTAGAAAG	Reverse primer for amplification of *eno* gene
P109	CTTTCTACAACCTTAACAAATAATAGATGAATACATCCTATTCACAATCG AA	Forward primer for amplification of kan^R gene
P110	ATTCCCCTTCCATTTTCACCTGTAATTAGAAATCCCTTTGAGAATGT	Reverse primer for amplification of kan^R gene
P111	ACATTCTCAAAGGGATTTCTAATTACAGGTGAAAATGGAAGGGGAATCCCTTCCT	Forward primer for amplification of downstream homologous sequence for *pyk* deletion
P112	TCAATCAGCATAAAAAGTTTCATATTCCGCCCGGGAACAGAATCGGCTTACACCAGCC	Reverse primer for amplification of downstream homologous sequence for *pyk* deletion
P113	ACGTACATATGTAATGGGCCGT	Forward primer for identification of *pyk* deletion
P114	GATGATGATCCGCTTATTCT	Reverse primer for identification of *pyk* deletion
P115	GAGATCGGTACTTCGCGAATGCGTCGAGATTACATGTATGCAGGAAATGGACGTAA	Forward primer for amplification of upstream homologous sequence for *trpE* deletion

P116	AGTTTTGAGAGTATTTGATGTTTTTGTTCATTGC TCTCACTCCTTATGGCAAGGAGAAT	Reverse primer for amplification of upstream homologous sequence for *trpE* deletion
P117	ATTCTCCTTGCCATAAGGAGTGAGAGCAATGA ACAAAAACATCAAATACTCTCAAAACT	Forward primer for amplification of *erm* gene
P118	ACCGAGATAATGTGCATGACATGTGAAAAGGTT ATTTACGGCCGTTGAAAAGAAGGTAA	Reverse primer for amplification of *erm* gene
P119	TTACCTTCTTTTCAACGGCCGTAAATAACCTTT TCACATGTCATGCACATTATCTCGGT	Forward primer for amplification of downstream homologous sequence for *trpE* deletion
P120	TCGATATCCAAAAGTTCAGCAGTTGATTCCAAT TGTAGAAATCTGTTCATCAGCTTTAT	Reverse primer for amplification of downstream homologous sequence for *trpE* deletion
P121	GATGGCAGTATGTACCGGTA	Forward primer for identification of *trpE* deletion
P122	TCAGCCTCACCGGCAGTAAG	Reverse primer for identification of *trpE* deletion
P123	ATAAAGCTGATGAACAGATTTCTACAATTGGAA TCAACTGCTGAACTTTTGGATATCGA	Forward primer for amplification of upstream homologous sequence for deleting 163-255 nt of *csrA* gene
P124	ATTTGTTAATACAAAAATAAAAAAATCCTTATT CTTCCTGAATGGTCAAGTAAAT	Reverse primer for amplification of upstream homologous sequence for deleting 163-255 nt of *csrA* gene
P125	ATTTACTTGACCATTCAGGAAGAATAAGGATTT TTTTATTTTTGTATTAACAAAAT	Forward primer for amplification of downstream homologous sequence for deleting 163-255 nt of *csrA* gene
P126	TTAAAATAGAGAGCCTGGCACCAGGCTAGATT CCGTCTTGAGAGTTTTTAGAAGCCAT	Reverse primer for amplification of downstream homologous sequence for deleting 163-255 nt of *csrA* gene
P127	AATTATGAATTTGATCTTGAT	Forward primer for identification of *csrA* inactivation
P128	TGCACCCTCAGCTGTTTGGATA	Reverse primer for identification of *csrA* inactivation
P129	ATGGCTTCTAAAAACTCTCAAGACGGAATCTA GCCTGGTGCCAGGCTCTCTATTTTAA	Forward primer for amplification of upstream homologous sequence for *pheA* deletion
P130	TTGTTCGTATGTATTCAAATATATCCTCCTCATG ACGATTTTCTCTCCCTTATGCA	Reverse primer for amplification of upstream homologous sequence for *pheA* deletion
P131	TGCATAAGGGAGAGAAAATCGTCATGAGGAGG ATATATTTGAATACATACGAACAA	Forward primer for amplification of $Spec^R$ gene
P132	TCCGTACACTTCGTTTACTTCCGCATTATAATTT TTTTAATCTGTTATTTAAATAGT	Reverse primer for amplification of $Spec^R$ gene
P133	ACTATTTAAATAACAGATTAAAAAAATTATAATG CGGAAGTAAACGAAGTGTACGGA	Forward primer for amplification of promoter P_{spoVG}
P134	AGAAGTTCAAGTTCTGTGTTAGACATCTATATA AAAGCATTAGTGTATCAATTCC	Reverse primer for amplification of promoter P_{spoVG}
P135	GGAATTGATACACTAATGCTTTTATATAGATGTC TAACACAGAACTTGAACTTCT	Forward primer for identification of $aroA^{C575T/G576T}$ integration
P136	GACTAAAAAAAGCCCTCTAGTGGGCTTTTTAG CGTTAACTTTAACCATAGGTTT	Reverse primer for identification of $aroA^{C575T/G576T}$ integration
P137	AAACCTATGGTTAAAGTTAACGCTAAAAAGCC CACTAGAGGGCTTTTTTTAGTC	Forward primer for amplification of downstream homologous sequence for *pheA* deletion

P138	TTCTGATTTGACTTGGATAACCCAGGCTTCTAAATCACCGCTGACAGGAGGAGAACT	Reverse primer for amplification of downstream homologous sequence for *pheA* deletion
P139	TCAATGTTGGCTGAAAAGAG	Forward primer for identification of *pheA* deletion and *aroA*$^{C575T/G576T}$ integration
P140	TCTTCTGTGGCTGAAGGAAT	Reverse primer for identification of *pheA* deletion and *aroA*$^{C575T/G576T}$ integration
P141	AGTTCTCCTCCTGTCAGCGGTGATTTAGAAGCCTGGGTTATCCAAGTCAAATCAGAA	Forward primer for amplification of upstream homologous sequence for *aroH* mutation (T261A/G262A/C263A / A268T/G269C)
P142	ATCATGACTGATATTTTCTTTTTCAGACCGCCTGTTA	Reverse primer for amplification of upstream homologous sequence for *aroH* mutation
P143	TGAAAAAGAAAATATCAGTCATGATGACGGTCCAGACAGATGT	Forward primer for amplification of downstream homologous sequence for *aroH* mutation
P144	GTGAACAAGGCTAGCTGCTACAATATACACTCTTGTGTATTTCTA	Reverse primer for amplification of downstream homologous sequence for *aroH* mutation
P145	ACGTTTGGTCAGCTGGCTA	Forward primer for identification of *aroH* mutation
P146	TTCATTGCTCTCACTCCTTA	Reverse primer for identification of *aroH* mutation
P147	TGCTGCATACATCCGCAACGAGGTTGCAAACGAATATTGTAGCAGCTAGCCTTGTTCAC	Forward primer for amplification of upstream homologous sequence for *tyrA* mutation (T915A/C927G/G933A/T936A/T937G/C939G/G948A/T951G)
P148	TGCGCTCCGCAGCTAAGATCGCTGTTATCTCCGCTATTACACCCGGATGATCGGGTACA	Reverse primer for amplification of upstream homologous sequence for *tyrA* mutation
P149	TGTACCCGATCATCCGGGTGTAATAGCGGAGATAACAGCGATCTTAGCTGCGGAGCGCA	Forward primer for amplification of downstream homologous sequence for *tyrA* mutation
P150	AACCGGCTTTGAATATCTCTTTAACAGGGCATCGATTCCTTTTCCGTGAATCACGACAT	Reverse primer for amplification of downstream homologous sequence for *tyrA* mutation
P151	TTACAAGCGTAATCAGTCAT	Forward primer for identification of *tyrA* mutation
P152	TCTAAAAGGCTTTCTGGCTCT	Reverse primer for identification of *tyrA* mutation
P153	ATGTCGTGATTCACGGAAAAGGAATCGATGCCCTGTTAAAGAGATATTCAAAGCCGGTT	Forward primer for amplification of upstream homologous sequence for *aroA* mutation (C575T/G576T)
P154	TAGTCCAGCGCTTCTTCGATATGAGCAAGAGTTACGATTTCACTGATAACCGC	Reverse primer for amplification of upstream homologous sequence for *aroA* mutation
P155	GCGGTTATCAGTGAAATCGTAACTCTTGCTCATATCGAAGAAGCGCTGGACTA	Forward primer for amplification of downstream homologous sequence for *aroA* mutation
P156	CGCCTCTGCACTCGTCGGTCCCGGCATCCGATAGTCAGAAAGTGCGACTGACGGATCA	Reverse primer for amplification of downstream homologous sequence for *aroA* mutation
P157	TGTTAAACAATATCATTGAA	Forward primer for identification of *aroA* mutation
P158	AGGAATCGCCATTTGCTGAGCA	Reverse primer for identification of *aroA* mutation
P159	TTCCAGAGATTGGGCCCACGCGTCCATGGGGTACAGCCTGGTGCCAGGCTCTCTATT	Forward primer for amplification of upstream homologous sequence for carless knocking *Spec*^{R} gene

P160	TCCGTACACTTCGTTTACTTCCGCAGACGATTT TCTCTCCCTTATGCA	Reverse primer for amplification of upstream homologous sequence for carless knocking *Spec*^{R} gene
P161	TGCATAAGGGAGAGAAAATCGTCTGCGGAAGT AAACGAAGTGTACGGA	Forward primer for amplification of downstream homologous sequence for carless knocking *Spec*^{R} gene
P162	AATGCAGCTGTGGAAATCGATGCATGCGCTAG CGCTATATAAAAGCATTAGTGTATCAA	Reverse primer for amplification of downstream homologous sequence for carless knocking *Spec*^{R} gene
P163	TTCCAGAGATTGGGCCCACGCGTCCATGGGGT ACACATGTATGCAGGAAATGGACGTAA	Forward primer for amplification of upstream homologous sequence for carless knocking *Erm*^{R} gene
P164	ACCGAGATAATGTGCATGACATGTGAAAAGGT GCTCTCACTCCTTATGGCAAGGAGAAT	Reverse primer for amplification of upstream homologous sequence for carless knocking *Erm*^{R} gene
P165	ATTCTCCTTGCCATAAGGAGTGAGAGCACCTTT TCACATGTCATGCACATTATCTCGGT	Forward primer for amplification of downstream homologous sequence for carless knocking *Erm*^{R} gene
P166	AATGCAGCTGTGGAAATCGATGCATGCGCTAG CGCAATTGTAGAAATCTGTTCATCAG	Reverse primer for amplification of downstream homologous sequence for carless knocking *Erm*^{R} gene
P167	TTCCAGAGATTGGGCCCACGCGTCCATGGGGT ACAGCTCTTCAAACAATCGTTGAAGCT	Forward primer for amplification of upstream homologous sequence for carless knocking *Kan*^{R} gene
P168	CTTCCATTTTCACCTGTAACTATTATTTGTTAAG GTTGTAGAAAG	Reverse primer for amplification of upstream homologous sequence for carless knocking *Kan*^{R} gene
P169	CTTTCTACAACCTTAACAAATAATAGTTACAGG TGAAAATGGAAGGGGAATCCCTTCCT	Forward primer for amplification of downstream homologous sequence for carless knocking *Kan*^{R} gene
P170	AATGCAGCTGTGGAAATCGATGCATGCGCTAG CGATTTGAACAGAATCGGCTTACA	Reverse primer for amplification of downstream homologous sequence for carless knocking *Kan*^{R} gene
P171	TTCCAGAGATTGGGCCCACGCGTCCATGGGGT AC	Forward primer for identification of resistant genes
P172	AATGCAGCTGTGGAAATCGATGCATGCGCTAG CG	Reverse primer for identification of resistant genes
P173	GTCTCGAGGCCTCCAGGCATGCAAGCTTGGCG TAATCAT	Forward primer for deletion of *Bgl*II and *Pst*I in the pHCMC04
P174	GAGGCCTCGAGACCTGGAAAAACAAATCGAG TTGTTAA	Reverse primer for deletion of *Bgl*II and *Pst*I in the pHCMC04
P175	TAGTGATATCTAAAAATCAAAGGGGGAAATGTC TACAACACTTATCCTTACAGGCGAA	Forward primer for amplification of optimized *tal* gene
P176	CGGGCTGCCCCGGGGACGTCGACTCTAGAGGA TCCAAATAATAAAAAAGCCGGATTAA	Reverse primer for amplification of optimized *tal* gene
P177	AGCCAGATTATTAATCCGGCTTTTTTATTATTTG TTAAAGGCCGTTAGCAAGTTTAGCA	Forward primer for amplification of optimized P_{veg}-*4cl* gene
P178	GGCTGCCCCGGGGACGTCGACTCTAGAGGATC CGGAGTTCTGAGAATTGGTATGCCTT	Reverse primer for amplification of optimized P_{veg}-*4cl* gene
P179	TGAGCGGGCTTTTTTCACGTCACGCGTCCATGG ATATTGAGTGGATGATTATATTCCT	Forward primer for amplification of optimized P_{43}-*sts* gene
P180	AAGGTATAAACTTTTCAGTTGCAGACAAAG ATCACCAGGCATCAAATAAA ACGAA	Reverse primer for amplification of optimized P_{43}-*sts* gene

Table S3 Accession numbers and sources of genes for constructing pMGR6, pMGR10, pMGR15, and pMGR-tyr10.

Genes	Sources	Accession numbers	Nucleotides
deoD	*Bacillus subtilis*	NC_000964.3	2135470-2136171
upp	*B. subtilis*	NC_000964.3	3788426-3789055
rpoB	*B. subtilis*	NC_000964.3	121919-125500
amyE	*B. subtilis*	NC_000964.3	327618-329597
purA	*B. subtilis*	NC_000964.3	4155433-4156725
thrC	*B. subtilis*	NC_000964.3	3313770-3314828
divIVA	*B. subtilis*	NC_000964.3	1612521-1613015
recU	*B. subtilis*	NC_000964.3	2340802-2341422
tetL	*B. subtilis*	NC_000964.3	4186607-4191637
trpC	*B. subtilis*	NC_000964.3	2374139-2374888
hisD	*B. subtilis*	NC_000964.3	3586271-3587554
metA	*B. subtilis*	NC_000964.3	2283634-2328027
lysA	*B. subtilis*	NC_000964.3	2436947-2438266
ilvA	*B. subtilis*	NC_000964.3	2292769-2294037
aprE	*B. subtilis*	NC_000964.3	1104423-1105568
tkt	*B. subtilis*	NC_000964.3	1919861-1921864
eno	*B. subtilis*	NC_000964.3	3476555-3477847
pyk	*B. subtilis*	NC_000964.3	2984788-2986545
aroA	*B. subtilis*	NC_000964.3	3045445-3046521

aroH	*B. subtilis*	NC_000964.3	2379100-2380272
trpE	*B. subtilis*	NC_000964.3	2375869-2377416
tyrA	*B. subtilis*	NC_000964.3	2369251-2370366
pheA	*B. subtilis*	NC_000964.3	2851283-2852140
csrA	*B. subtilis*	NC_000964.3	3636046-3636270

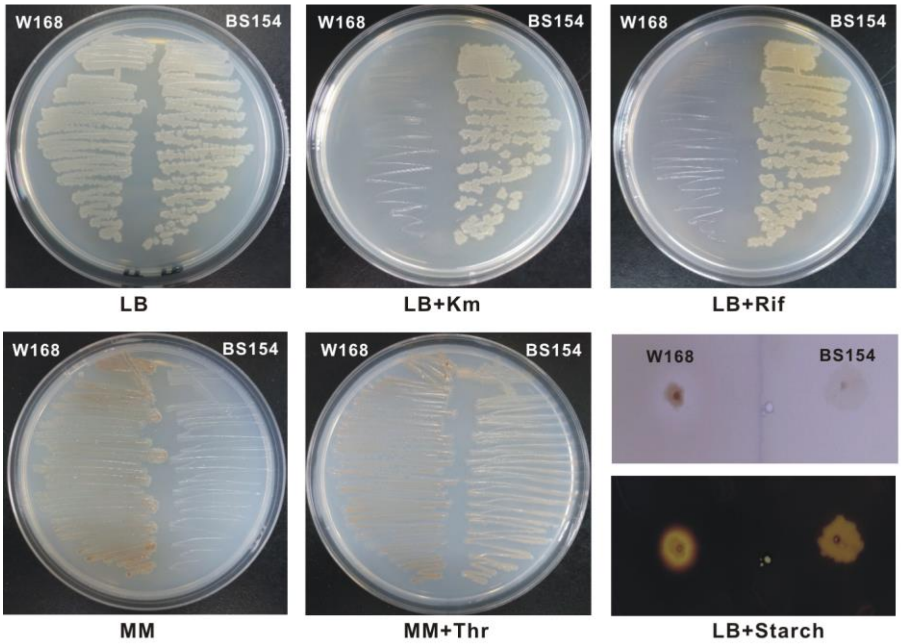

Figure S1. Verification of the quadruple mutant BS155 (WYB108$\triangle$*deoD::kan*^{R} *ropB* amyE* thrC**) by selective plates. The *deoD*-, *ropB*-, and *amyE*-inactive strains were verified by LB plates containing kanamycin (LB-Km), rifampicin (LB-Rif), and starch, respectively. The *thrC*-inactive strain was verified by MM plates with or without threonine (Thr).

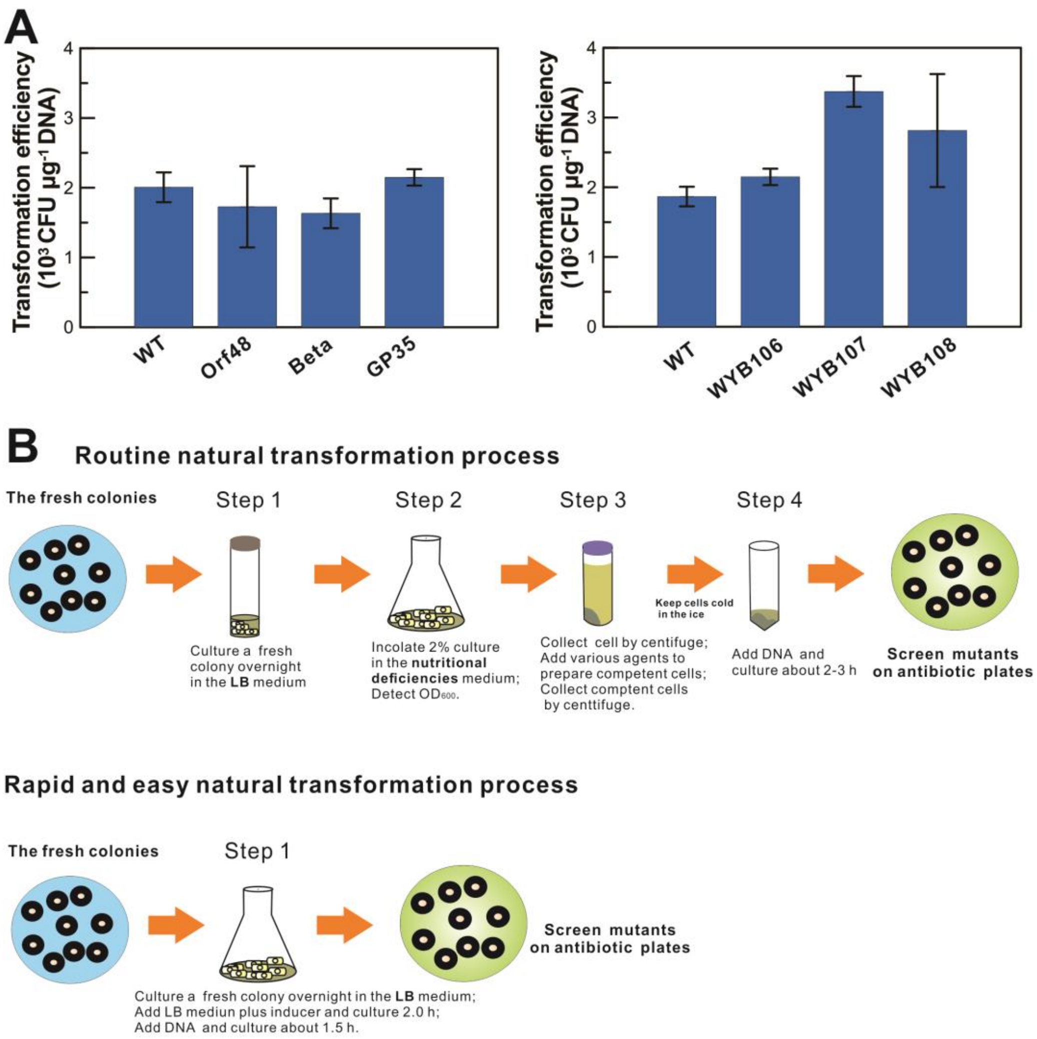

Figure S2. (A) The transformation efficiencies of the high recombinant strains expressing the recombinase or/and competency factor. All error bars indicate ±SD, n=3. (B) The overall processes of the natural transformation in the super competent cell are more rapid and easy compared to the routine natural transformation. Complex nutritional deficiencies medium need to be prepared in the transitional natural transformation, such as MD medium consisted of 9.29 g l^{-1} K_2HPO_4, 5.33 g l^{-1} KH_2PO_4, 0.78 g l^{-1} Trisodium citrate, 20 g l^{-1} Glucose, 0.05 g l^{-1} L-tryptophan, 0.01 g l^{-1} ferric ammonium citrate, 2 g l^{-1} potassium aspartate, and 0.36 g l^{-1} $MgSO_4$.

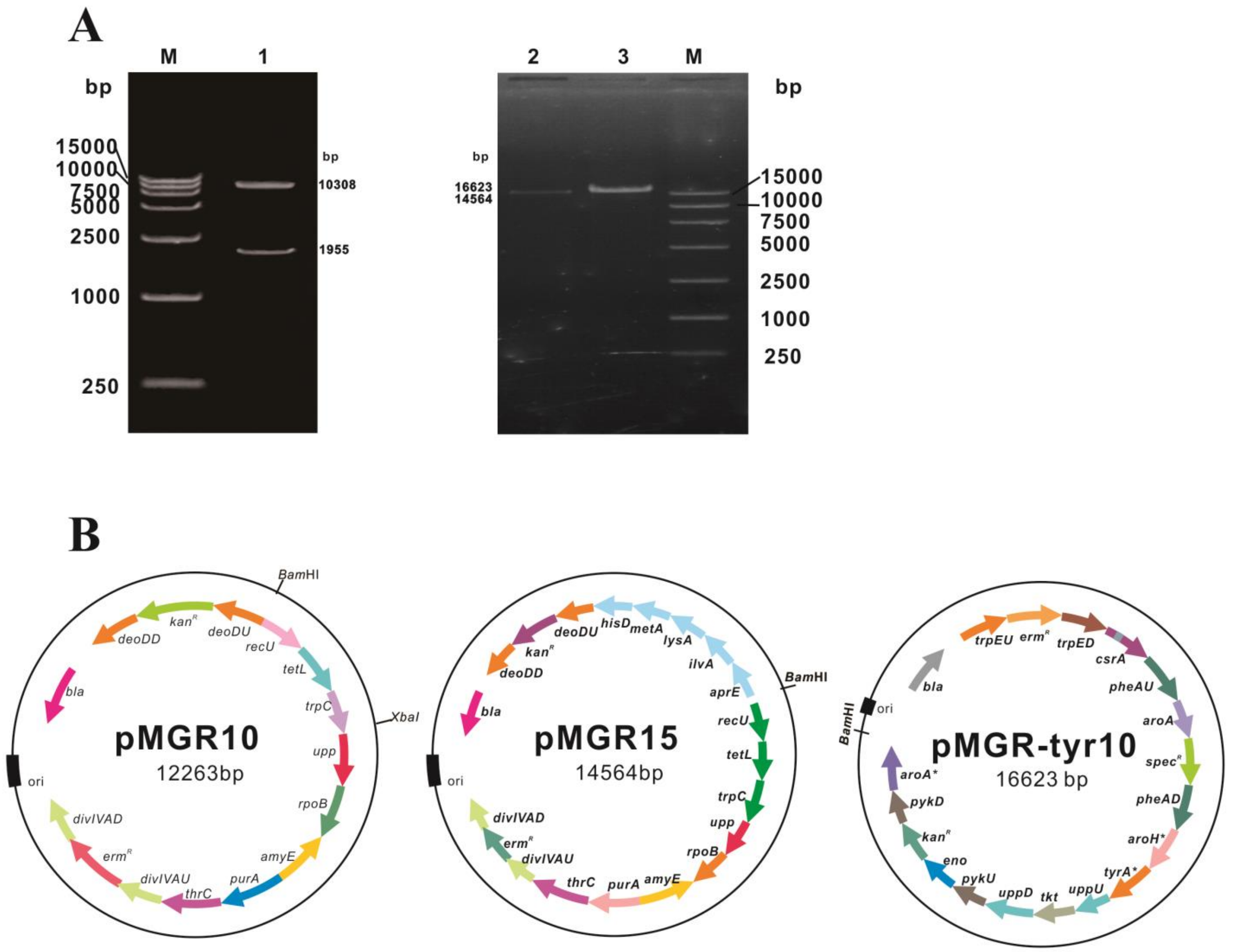

Figure S3. The construction of the all-in-one recombination plasmids. (A) The verification of plasmids pMGR10, pMGR15, and pMGR-tyr10 digested with restriction enzymes. The plasmid pMGR10 was verified by digestion with *Bam*HI and *Xba*I (Lane 1). Plasmids pMGR15 (Lane 2) and pMGR-tyr10 (Land 3) were separately verified by digestion with *Bam*HI. (B) The maps of plasmids pMGR10, pMGR15, and pMGR-tyr10. The detailed information of plasmid construction was shown in Supplementary Methods.

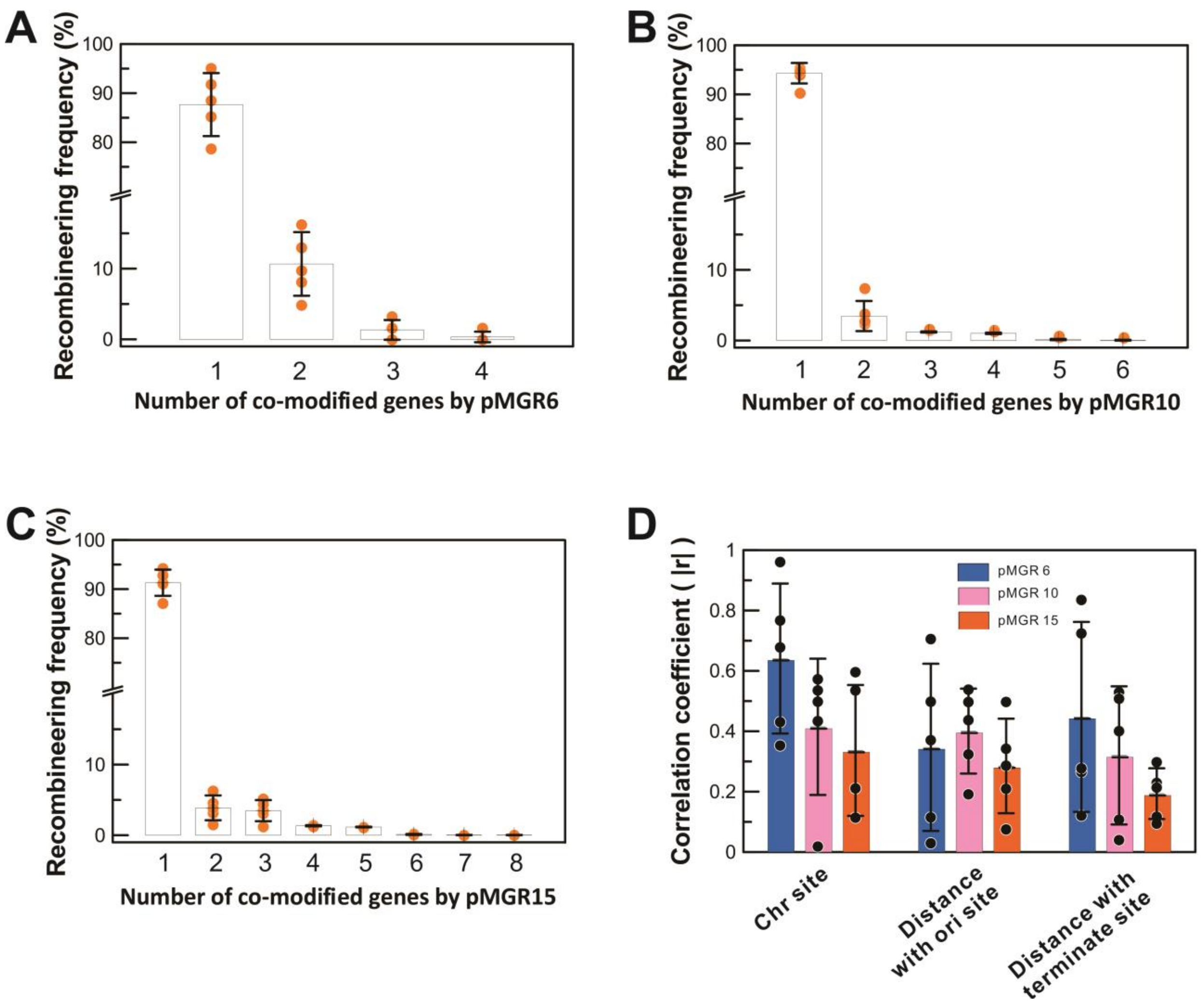

Figure S4. The editing frequency for co-modified variants and correlation using the SMGE Method. (A)-(C) Co-editing frequencies of multiplex variants using different pMGR vectors. (D) The correlation coefficients of recombineering frequencies for each target with chromosome site (Chr site), distance of ori site and terminate site. In statistics, the correlation coefficient r is used to measure the strength and direction of a linear relationship between two variables on a scatterplot. |r| represents the correlation coefficient. The value of |r| is always between 0 and +1. |r|=1 means a perfect downhill (negative) linear relationship. 0.70≦ |r| < 1 means a strong downhill (negative) linear relationship. 0.50 ≦|r| < 0.70 means a moderate downhill (negative) relationship. 0.30≦|r|< 0.50 means weak downhill (negative) linear relationship. |r|< 0.30 means no linear relationship. The dot represents five repeated data by the transformation of pMGR6, pMGR10, and pMGR15.

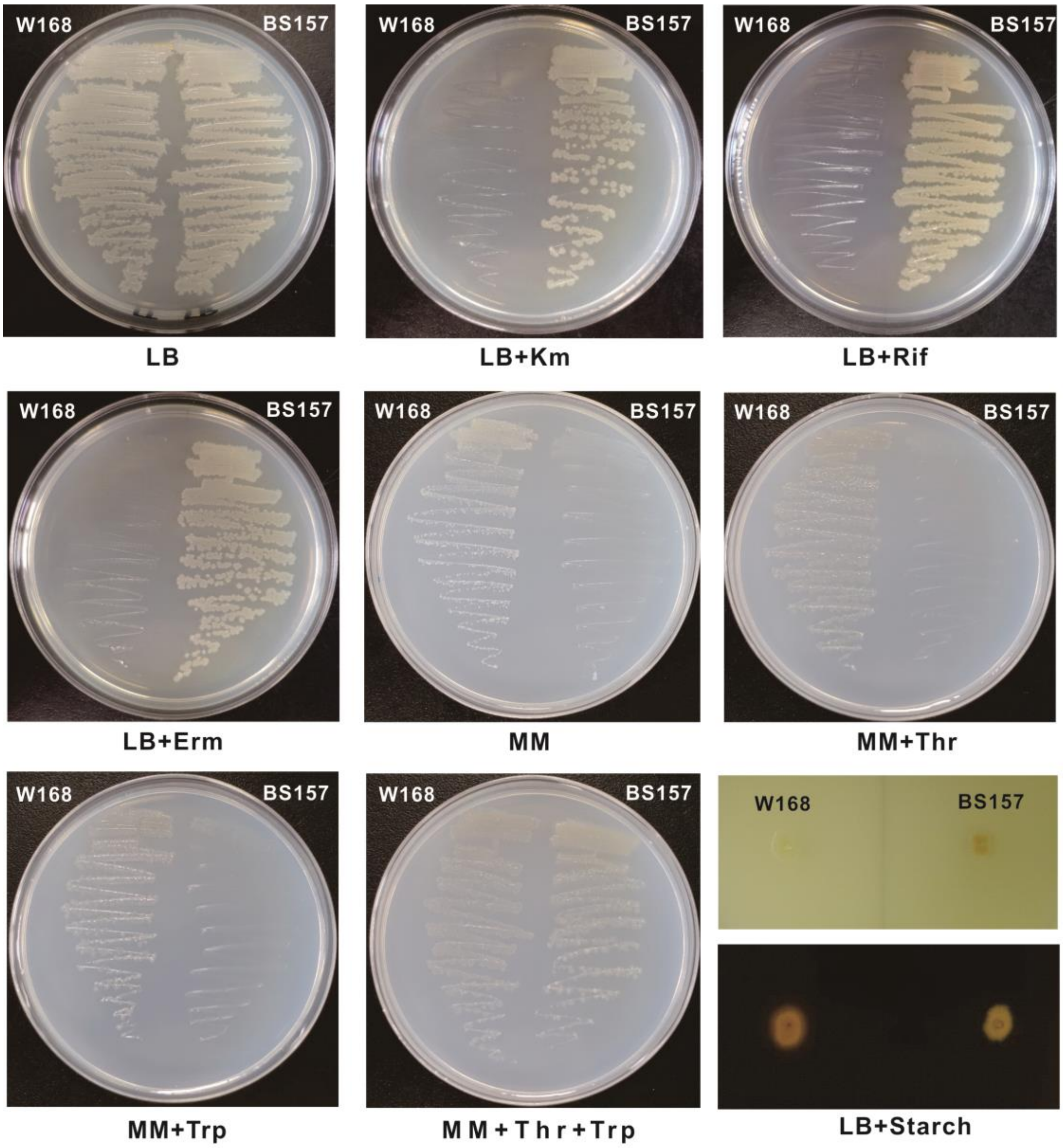

Figure S5. The sextuple mutant BS157 (WYB108 Δ*divIVA::erm*^{R} Δ*deoD::kan*^{R} *rpoB** *amyE** *trpC** *thrC**) were verified by selective plates. The *deoD*-, *rpoB*-, *divIVA*-, and *amyE*-inactive strains were verified by LB plates containing kanamycin (LB-Km), rifampicin (LB-Rif), erythromycin (LB-Erm), and starch, respectively. The *thrC*- and *trpC*-inactive strains were separately verified by MM plates with or without threonine (Thr) and tryptophan (Trp).

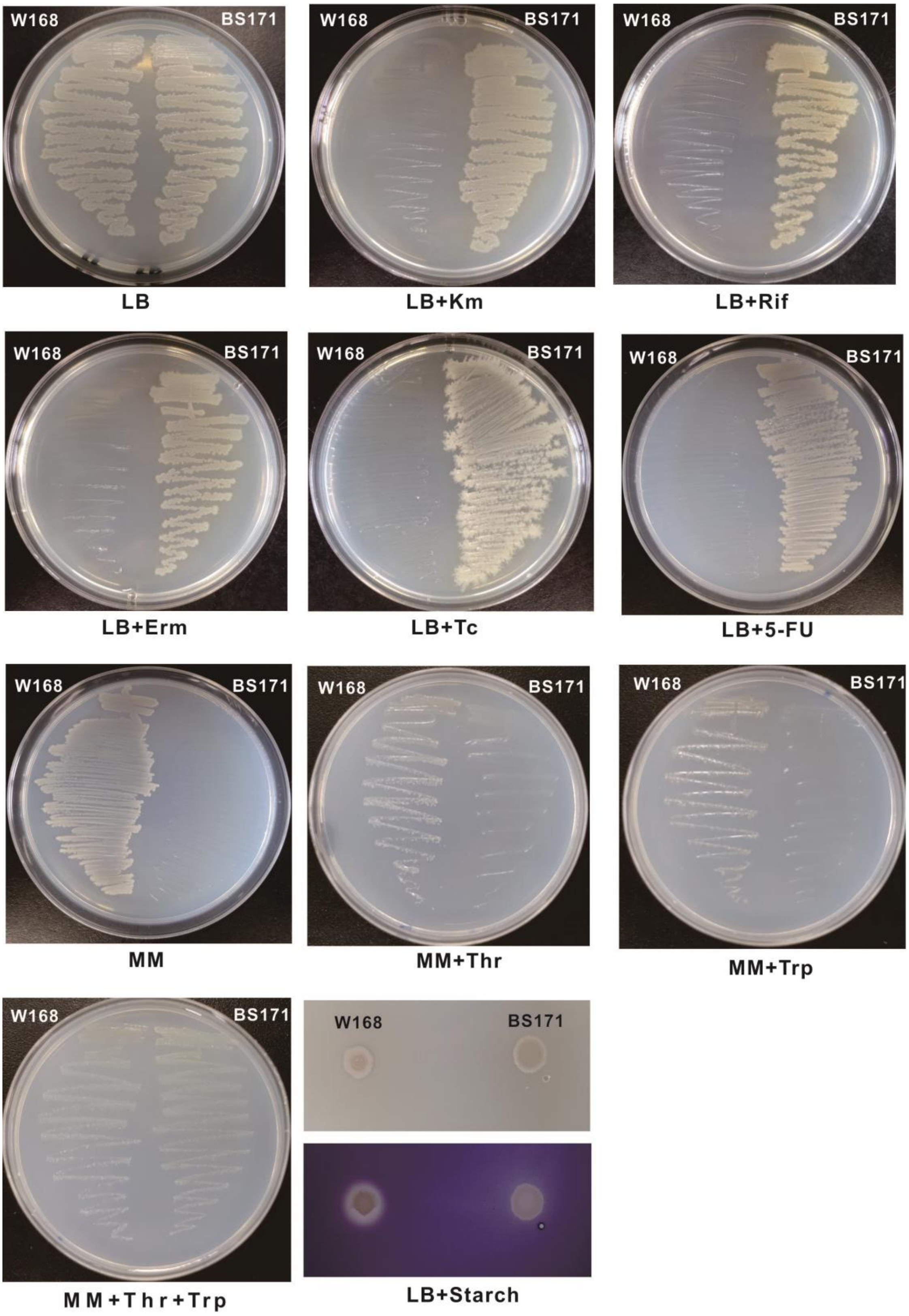

Figure S6. The octuplet mutant BS171 (WYB108 Δ*divIVA::erm*^{R} Δ*deoD::kan*^{R} *rpoB* amyE* upp* tetL* thrC* trpC**) were verified by selective plates. The *deoD*-, *ropB*-, *divIVA*-, *tetL*-, and *amyE*-inactive strains were verified by LB plates containing kanamycin (LB-Km), rifampicin (LB-Rif), erythromycin (LB-Erm), tetracycline (LB-Tc) and starch, respectively. The *thrC*- and *trpC*-inactive strains were separately verified by MM plates with or without threonine (Thr) and tryptophan (Trp).

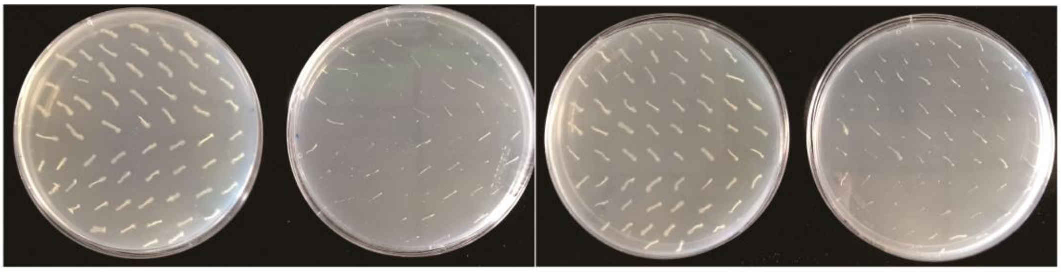

Figure S7. Selection and verification of variants with optimized tyrosine biosynthesis. (A) Verification of variants that eliminated the GP35 and ComKS expression vector. Up to 450 variants were randomly selected on selective plates containing kanamycin, erythromycin, or spectinomycin after the transformation of pMGR-tyr10. They were inoculated successively for 9 generations (3 days) in 96-well plates containing LB media to eliminate pWYE897 (pHCMC04-*comKS*-*gp35*). Variants that could not grow on the LB-Cm plate were determined to eliminate the vector.

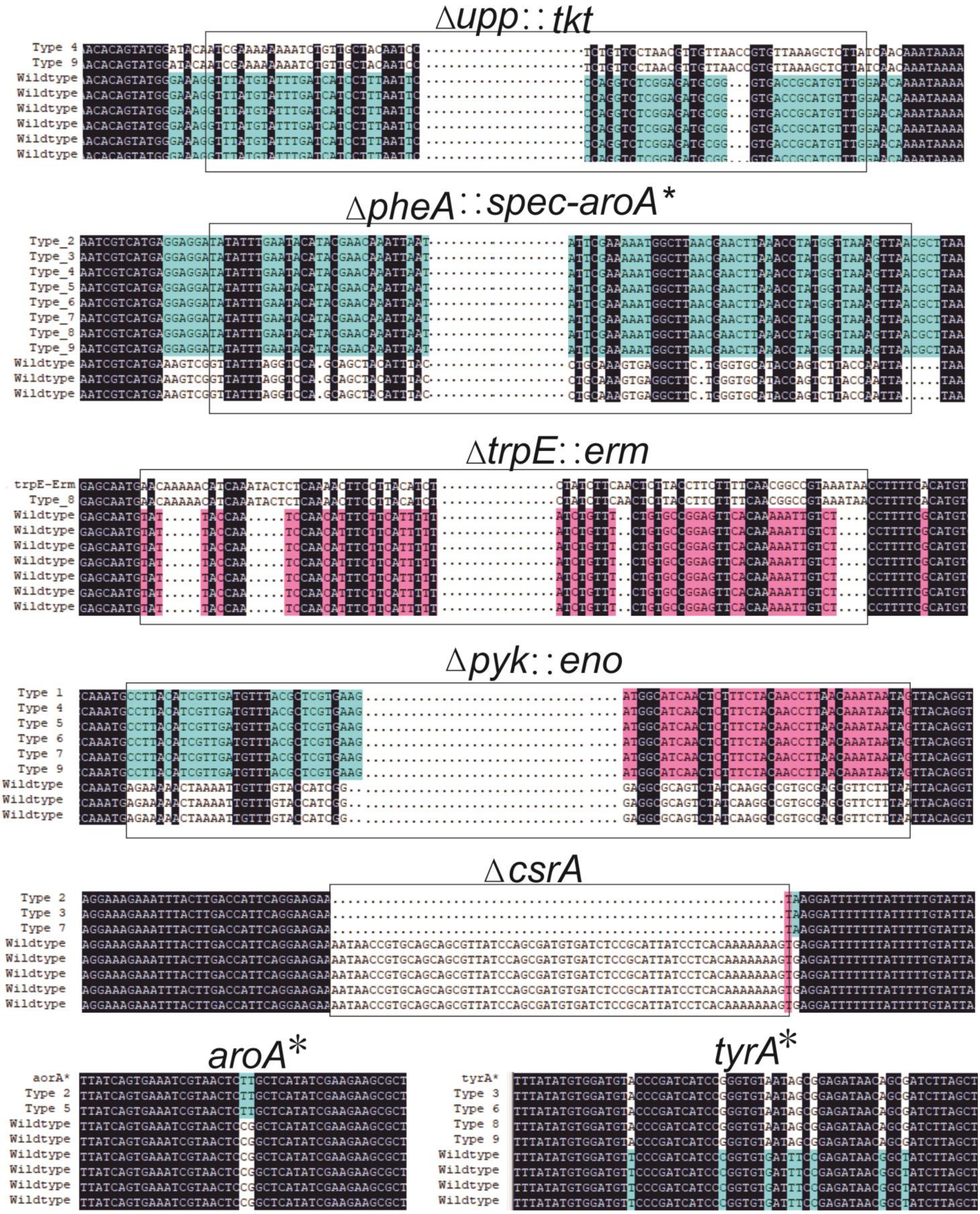

Figure S8. Verification of mutation by sequence alignments. After selected from 48-well plates (Figure 6b), 26 variants exhibited 4- to 10-fold increases in tyrosine production, which were sequenced and aligned to determine mutant sites. The correct mutation sites were determined by PCR and DNA sequencing using a set

of primers binding upstream or downstream of homologous sequences. The bases highlighted in dark are the same to that of wild-type sequences, while the mutant bases were shown in pink or green.

References

Gibson, D.G., Young, L., Chuang, R.Y., Venter, J.C., Hutchison, C.A., and Smith, H.O. (2009). Enzymatic assembly of DNA molecules up to several hundred kilobases. *Nat. Methods* 6(5)**,** 343-U341. doi: 10.1038/nmeth.1318.

GueroutFleury, A.M., Shazand, K., Frandsen, N., and Stragier, P. (1995). Antibiotic-resistance cassettes for *Bacillus subtilis*. *Gene* 167(1-2)**,** 335-336. doi: 10.1016/0378-1119(95)00652-4.

Kunst, F., Ogasawara, N., Moszer, I., Albertini, A.M., Alloni, G., Azevedo, V., et al. (1997). The complete genome sequence of the Gram-positive bacterium *Bacillus subtilis*. *Nature* 390(6657)**,** 249-256. doi: 10.1038/36786.

Li, H.J., Zhang, G.Q., Deng, A.H., Chen, N., and Wen, T.Y. (2011). De novo engineering and metabolic flux analysis of inosine biosynthesis in *Bacillus subtilis*. *Biotechnol. Lett. 33*, 1575-1580. doi: 10.1007/s10529-011-0597-5.

Sun, Z.P., Deng, A.H., Hu, T., Wu, J., Sun, Q.Y., Bai, H., et al. (2015). A high-efficiency recombineering system with PCR-based ssDNA in *Bacillus subtilis* mediated by the native phage recombinase GP35. *Appl. Microbiol. Biotechnol.* 99(12)**,** 5151-5162. doi: 10.1007/s00253-015-6485-5.

Turgay, K., Hahn, J., Burghoorn, J., and Dubnau, D. (1998). Competence in *Bacillus subtilis* is controlled by regulated proteolysis of a transcription factor. *Embo J.* 17(22)**,** 6730-6738. doi: 10.1093/emboj/17.22.6730.

Wu, J., Deng, A., Sun, Q., Bai, H., Sun, Z., Shang, X., et al. (2018). Bacterial genome editing via a designed toxin-antitoxin cassette. *ACS Synth. Biol.* 7(3)**,** 822-831. doi: 10.1021/acssynbio.6b00287.

Zhang, G.Q., Wang, W.Z., Deng, A.H., Sun, Z.P., Zhang, Y., Liang, Y., et al. (2012). A Mimicking-of-DNA-Methylation-Patterns pipeline for overcoming the restriction barrier of bacteria. *PLoS Genet.* 8(9)**,** e1002987. doi: e1002987.10.1371/journal.pgen.1002987.
